# Targeting WDPF domain of Hsp27 achieves a broad spectrum of antiviral

**DOI:** 10.1002/mco2.70032

**Published:** 2025-02-26

**Authors:** Mandi Wu, Wei Li, Houying Leung, Yiran Wang, Qianya Wan, Peiran Chen, Cien Chen, Yichen Li, Xi Yao, Ming‐Liang He

**Affiliations:** ^1^ Department of Biomedical Sciences City University of Hong Kong Hong Kong Special Administrative Region Hong Kong China; ^2^ Weihai Municipal Hospital Cheeloo College of Medicine Shandong University Weihai Shandong China; ^3^ CityU Shenzhen Research Institute Nanshan Shenzhen China

**Keywords:** antiviral, hnRNP A1 translocation, Hsp27, phosphorylation, WDPF domain

## Abstract

Enterovirus A71 (EV‐A71) is a positive‐sense single‐stranded RNA virus, which hijacks host proteins to benefit viral internal ribosome entry site (IRES)‐dependent protein translation and further propagation. We demonstrated that serine 78 (S78) phosphorylation of Hsp27 is critical for Hsp27/hnRNP A1 relocalization upon EV‐A71 infection. Here, we report that the deletion of WDPF and ACD domains disturbs subcellular localization of Hsp27, resulting in partial nuclear translocation. The domain deletion‐induced Hsp27 nuclear translocation fails to direct hnRNP A1 translocation. The 2A^pro^‐induced IRES activity and viral replication are suppressed by the deletion of WDPF or ACD domain. Surprisingly, a peptide (WDPF) dramatically inhibits S78 phosphorylation. Therefore, hnRNP A1 translocation, viral IRES activity, and viral protein translation and propagation are all strongly suppressed by the WDPF peptide, but not by peptide without WDPFR sequence (ΔWDPF). Moreover, the WDPF peptide also has potent antiviral activity on other RNA virus (e.g., coronavirus HCoV‐OC43) and DNA virus (e.g., HSV‐1 and HBV). Peptide treatment with kinase inhibitor Sorafenib brings an additional inhibitory effect on HCoV‐OC43 and HSV‐1. Taken together, we uncover a crucial role of WDPF domain in S78 phosphorylation for EV‐A71‐induced hnRNP A1 nuclear translocation, IRES‐dependent viral protein translation, and EV‐A71 propagation. Our results explore a new path for target‐based pan‐antiviral strategy.

## Graphical abstract

Ser78 phosphorylation of Hsp27 plays an important role in Hsp27 nuclear translocation, which induces hnRNP A1 cytosol relocation and further enhances the IRES activity and viral replication mediated by EV‐A71 2A^pro^. Hsp27‐derived WDPF peptide competitively inhibited Hsp27 phosphorylation on serine 78 to block Hsp27/hnRNP A1 translocation and further IRES‐dependent viral reproduction.

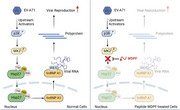

## 1 INTRODUCTION

Enterovirus A71 (EV‐A71) is a positive‐sense single‐stranded RNA virus [(+) ssRNA)] in the Picornavirus family. EV‐A71 infection can cause hand, foot, and mouth disease (HFMD) in children under 5 years old.^1^, ^2^ Although HFMD is self‐limiting and mild in most cases, a small portion of patients suffers from fatal neurological diseases, including aseptic meningitis, rhombencephalitis, acute flaccid paralysis, and brainstem encephalitis.^3^, ^4^ In recent years, large‐scale outbreaks of EV‐A71‐caused HFMD cases have been reported in China, Vietnam, South Korea, Russia, and Singapore.^5^, ^6^ In China, an EV‐A71 C4‐genotype‐based vaccine is the only licensed vaccine in the world.^7^, ^8^, ^9^, ^10^ However, supportive therapy remains the primary treatment, as there are no approved effective antiviral drugs for EV‐A71 infection.^11^, ^12^

The EV‐A71 genome encodes an open reading frame for a polyprotein, which is proteolytically cleaved into four structural proteins and seven nonstructural proteins.^13^, ^14^ The four structural proteins, VP1, VP2, VP3, and VP4, assemble to form the viral capsid, which is responsible for viral receptor binding, entry, stability, and assembly.^15^ Nonstructural proteins are 2A^pro^, 2B, 2C, 3A, 3B (also known as VPg), 3C^pro^, and 3D^pol^, which are associated with viral uncoating, protein processing, RNA replication, and immune evasion.^16^, ^17^, ^18^, ^19^, ^20^ For example, the 2A protease (2A^pro^) cleaves the newly synthesized viral polyprotein into functional proteins, suppresses host cap‐dependent translation, and induces internal ribosome entry site (IRES)‐dependent translation.^21^, ^22^

To initiate the polyprotein translation, EV‐A71 utilizes an IRES element, located in 5′UTR, in a cap‐independent manner, with the help of host IRES‐specific transacting factors (ITAFs). ITAFs bind to the IRES elements to promote the ribosome recruitment. Numerals ITAFs are involved in regulating EV‐A71 infection, such as far‐upstream‐element binding protein 1 (FBP1), FBP2, heterogeneous nuclear ribonucleoprotein A1 (hnRNP A1), hnRNP K, and poly(rC)‐binding protein 1 (PCBP1).^23^ FBP1 facilitates EV‐A71 IRES activity, while FBP2 has an opposite, negative impact.^24^, ^25^ hnRNP K plays an essential role in viral protein synthesis.^26^ hnRNP A1 changes the conformation of IRES element to direct the initiation of viral translation.^27^, ^28^ The virus‐induced relocalization of hnRNP A1 has been reported to benefit viral infection in cytoplasm, which is further promoted by Rhinovirus 2A^pro^ and 3C^pro^.^29^ We previously reported that EV‐A71 hijacks heat shock protein 27 (Hsp27) to induce hnRNP A1 cytosolic translocation.^30^ Recently, we further demonstrated that serine 78 (S78) phosphorylation of Hsp27 is critical for EV‐A71‐ or 2A^pro^‐induced hnRNP A1 cytosolic translocation and IRES‐dependent viral protein translation.^31^

Hsp27, a chaperone protein, functions under various stimuli, including high temperature, chemical treatment, UV, radiation, and pathogen infections. Hsp27 exists as monomers, dimers, and oligomers in cells, which are regulated by the phosphorylation at serine 15, 78, 82 upon signal transductions.^32^ Phosphorylation of Hsp27 is induced by mitogen‐activated protein kinases associated protein kinases (MAPKAP kinase 2, MK2), a downstream factor of MAP p38 protein kinase.^33^, ^34^ Hsp27 normally locates in cytoplasm, while phosphorylated Hsp27 is present in both cytoplasm and nucleus to respond to different stimuli.^35^, ^36^ Hsp27 is composed of a highly conserved α crystalline domain (ACD) domain, flanked by an N‐terminal WDPF domain and a flexible C‐terminal domain. The less conserved WDPF domain is associated with the chaperone activity of Hsp27.^37^ The flexible C‐terminal domain is involved in the regulation of protein interactions and oligomer solubility.^38^ The WDPF domain and C‐terminal domain can both interact with the ACD domain, changing the conformation to generate and stabilize oligomers by regulating the S78 and S82 phosphorylation.^39^, ^40^, ^41^, ^42^ Here, we report that WDPF domain plays a crucial role in S78 phosphorylation, and a peptide derived from the WDPF domain dramatically inhibits S78 phosphorylation and viral propagation in a dose‐dependent manner, paving the way for the development of effective EV‐A71 antivirals.

## 2 RESULTS

### 2.1 Critical roles of WDPF and ACD domains in Hsp27 cellular localization

**FIGURE 1:**
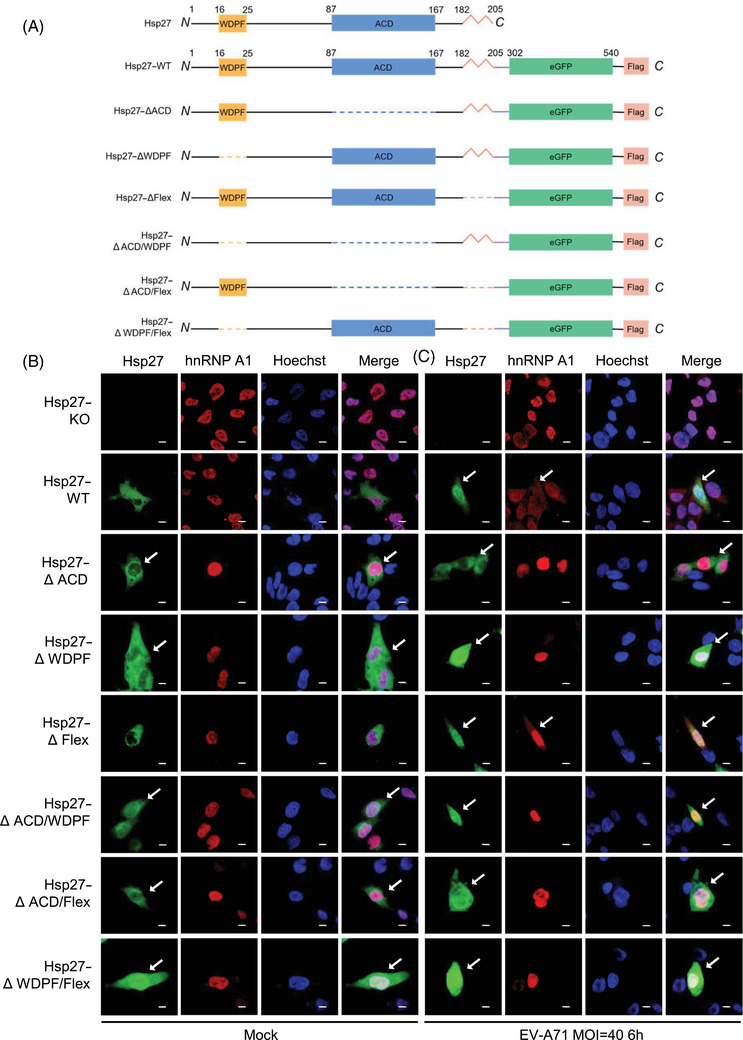
Blockage of hnRNP A1 nuclear translocation by WDPF domain and ACD domain truncation of Hsp27 upon EV‐A71 infection. (A) Diagram of Hsp27–eGFP and domain deletion constructs. Yellow, conserved α‐crystallin domain; blue, WDPF‐domain; orange zigzag line, flexible C‐terminal sequence; purple line, linker; green, eGFP; pink, Flag. The residue positions are indicated above the diagram. (B and C) Hsp27–KO cells were coinfected with Lenti–hnRNPA1–mCherry and Lenti–Hsp27–WT–eGFP–Flag (or Lenti–Hsp27–ΔACD–eGFP–Flag, Lenti–Hsp27–ΔWDPF–eGFP–Flag, Lenti–Hsp27–ΔFlex–eGFP–Flag, Lenti–Hsp27–ΔACD/∆WDPF–eGFP–Flag, Lenti–Hsp27–ΔACD/∆Flex–eGFP–Flag, Lenti–Hsp27–ΔWDPF/∆Flex–eGFP–Flag) for 3 days, then infected with or without EV‐A71 at the MOI of 40 for 6 h. The cells were fix and the nuclei were stained with Hoechst (blue). The images were captured by Nikon A1HD25 Confocal Microscope. For Hsp27, cells with nuclear translocation were marked by white arrows. For hnRNP A1, cells with cytosol translocation were marked by white arrows. The scale bar is 10 µM.

To explore the important domains governing EV‐A71‐induced Hsp27 translocation, we generated a series of Hsp27 truncation mutants by deleting one or two functional domains, including the ACD, the WDPF domain, and the flexible region (Flex) (Figure 1A). We stably expressed these Hsp27–eGFP fusion constructs in Hsp27 knockout (Hsp27–KO) RD cells using lentiviral transduction.^30^ The cells were infected with EV‐A71 at MOI 40 for 6 h. However, the protein expression levels of Hsp27 with ACD deletion (Hsp27∆ACD, Hsp27∆ACD/∆WDPF, and Hsp27∆ACD/∆Flex) are hardly detected by western blot in Hsp27–KO cells (Figure S1), suggesting a critical role of the ACD domain in Hsp27 protein stabilization. The nuclear localization of Hsp27 and its mutants was observed and captured under confocal microscopy. As shown in Figure 1B, Hsp27 and Hsp27∆Flex naturally localizes in the cytosol; Hsp27∆ACD shows certain nuclear localization and mostly cytosol localization, while WDFP deletion ensures a large portion of Hsp27 localizing in nuclei without EV‐A71 infection, indicating that WDFP domain (^16^WDPFRDWYPH^25^) plays an inhibitory role in Hsp27 nuclear localization. Alternatively, deletion of WDFP may generate an unknown, nonspecific nuclear localization signal (NLS). Consistently, simultaneous deleting WDFP domain with either ACD domain or Flex domain ensures Hsp27 to localize in both cytoplasm and nucleus; whereas less amount of Hsp27 protein is in the nucleus when the ACD and Flex regions were both deleted. Quantitative colocalization analysis revealed that the deletion of the ACD and WDPF domains significantly increased the Manders’ colocalization coefficient M1 between Hsp27 and the nuclear marker Hoechst, suggesting enhanced Hsp27 nuclear redistribution (Figure S2A). Upon EV‐A71 infection, wild‐type Hsp27 and all the truncation mutants exhibited similar patterns of nuclear translocation (Figures 1C and S2A). These demonstrated an important role of ACD domain and WDPF domains in Hsp27 subcellular localization.

### 2.2 Essential role of WDPF and ACD domains in hnRNP A1 translocation upon EV‐A71 infection

hnRNP A1–mCherry fusion protein was coexpressed in Hsp27–KO cells with the restoration of wild‐type or mutant Hsp27 constructs using lentiviral vectors to examine their subcellular localization. As expected, without any stimuli, hnRNP A1 did not translocate into cytoplasm when coexpressed with wild‐type Hsp27 or Hsp27 mutants lacking the ACD domain (Hsp27WT, Hsp27∆ACD, Hsp27∆ACD/Flex) (Figure 1B). Surprisingly, hnRNP A1 did not redistribute to the cytosol even though Hsp27 translocated into the nucleus when WDPF domain was deleted (Figure 1B; images from Hsp27∆WDFP, Hsp27∆ACD/∆WDFP, Hsp27∆WDFP/∆Flex), indicating that the nuclear Hsp27∆WDFP is not sufficient to induce the cytosolic redistribution of hnRNP A1.

Furthermore, the cells were infected with EV‐A71 at the MOI of 40 for 6 h to capture the subcellular localization of Hsp27 (or its domain‐deleted mutants) and hnRNP A1. The colocalization of hnRNP A1 and Hoechst was also quantified. Deletion of Flex region did not affect the cytosolic redistribution of hnRNP A1 (Figures 1C and S2B; Hsp27∆Flex), indicating that the cytosolic translocation of hnRNP A1 is independent of the Flex of Hsp27. Importantly, the cytosol redistribution of hnRNPA1 was blocked by the deletion of the ACD domain of Hsp27 (Figures 1C and S2B; Hsp27∆ACD). The absence of WDPF domain abolished the cytoplasmic translocation of hnRNP A1 completely, even though more Hsp27∆WDFP mutant translocated into the nucleus upon EV‐A71 infection (Figures 1C and S2B; Hsp27∆WDFP). Consistently, the simultaneous deletion of two domains of Hsp27 blocked the translocation of hnRNP A1 (Figures 1C and S2B; Hsp27∆ACD/∆WDPF, Hsp27∆ACD/∆Flex, Hsp27∆WDPF/∆Flex). Collectively, both ACD and WDPF domains are essential to EV‐A71‐induced cytosol hnRNPA1 translocation for IRES‐dependent translation.

**FIGURE 2:**
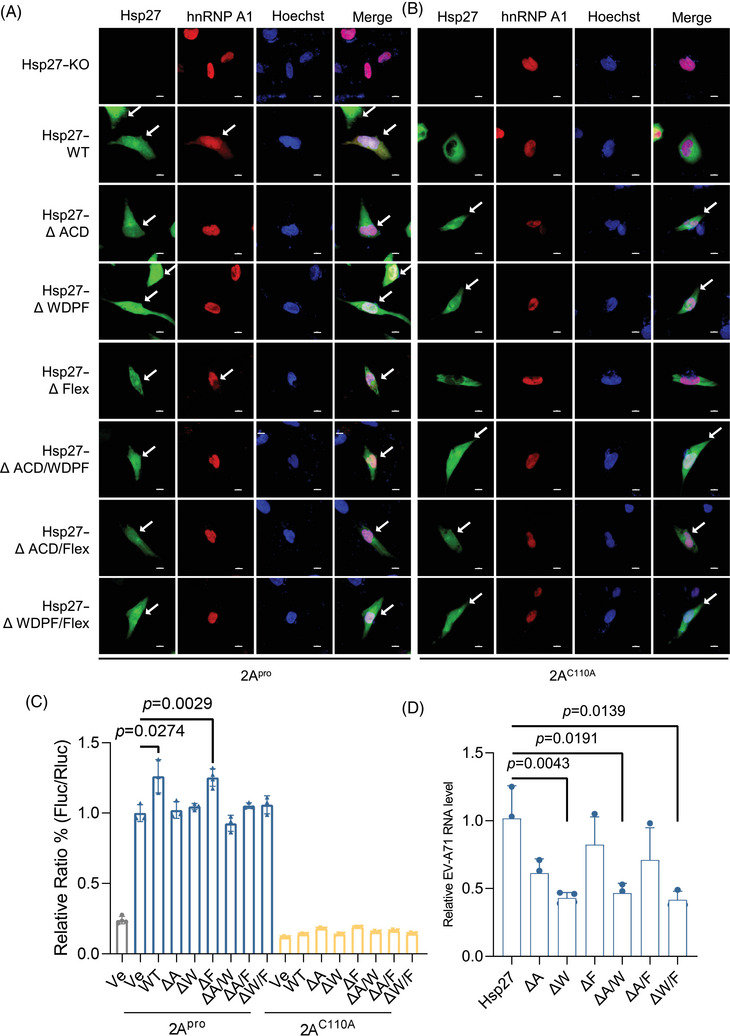
Inhibition of 2A^pro^‐induced hnRNP A1 redistribution and viral IRES‐dependent translation by ACD and WDPF domains truncation. (A and B) Hsp27–KO RD cells on the coverslips were coinfected with Lenti–hnRNPA1–mCherry and Lenti–Hsp27–WT–eGFP (or Lenti–Hsp27–ΔACD–eGFP, Lenti–Hsp27– ΔWDPF–eGFP, Lenti–Hsp27–ΔFlex–eGFP, Lenti–Hsp27–ΔACD/∆WDPF–eGFP, Lenti–Hsp27– ΔACD/∆Flex–eGFP, Lenti–Hsp27–ΔWDPF/∆Flex–eGFP) at the MOI of 20 for 3 days, then transfected with 2A^pro^ or 2A^C110A^ plasmids (500 ng) for 24 h. The cells were fixed, and the nuclei were stained with Hoechst (Blue). The images were captured by Nikon A1HD25 Confocal Microscope. For Hsp27, cells with nuclear translocation were marked by white arrows. For hnRNP A1, cells with cytosol translocation were marked by white arrows. The scale bar is 10 µM. (C) HEK293T cells were cotransfected with Hsp27 plasmids (WT, Hsp27–ΔACD, Hsp27–ΔWDPF, Hsp27–ΔFlex, Hsp27–ΔACD/∆WDPF, Hsp27–ΔACD∆/Flex, Hsp27–ΔWDPF/∆Flex) plasmid (800 ng), 2A^pro^ or 2A^C110A^‐expressing plasmid (200 ng) and pIRES reporter plasmid (200 ng) for 24 h, then the luciferase activity was measured. (D) Hsp27–KO RD cells were infected with lentiviral vectors carrying Hsp27 WT and its truncations for 3 days, then infected with EV‐A71 at the MOI of 1 for 9 h. Intracellular viral RNA was extracted and quantified by qPCR. Statistical analyses were carried out using Student's *t*‐test. Data are expressed as mean ± SD.

The 2A^pro^‐induced Hsp27/hnRNP A1 relocalization was also examined. Consistent with EV‐A71 infection, after 2A^pro^ transfection, Hsp27 and its mutants localized both in nucleus and cytoplasm (Figures 2A and S2C). However, the translocation of hnRNP A1 was blocked by the deletion of WDPF domain or ACD domain (Figures 2A and S2D). Importantly, consistent with previous results, the translocation of Hsp27/hnRNP A1 was dependent on the protease activity as the inactive 2A^pro^ mutant (2A^C110A^) failed to direct the relocalization of Hsp27 or hnRNP A1 in the presence of Hsp27 or its Hsp27 mutants (Figures 2B and S2C,D).

### 2.3 Critical role of WDPF and ACD domains in IRES‐dependent translation and viral propagation

We further investigated the effect of three domains on 2A^pro^‐induced IRES activity. We cotransfected HEK293T cells with Hsp27 and its mutants, 2A^pro^‐expressing, and an IRES‐driven dicistronic reporter plasmids for 24 h. The renilla luciferase (Rluc) and firefly luciferase (Fluc) activities were measured using a dual‐luciferase assay kit. The normalized ratio of Fluc to Rluc represented the relative IRES activity. 2A^pro^ increased the IRES activity by approximately 10 folds (Figure 2C). In HEK293T cells, Hsp27–WT and Hsp27∆Flex increased IRES activity by 30% (Figure 2C). However, both Hsp27∆WDPF and Hsp27∆ACD failed to enhance the IRES activity. Consistently, all Hsp27 mutants with the deletion of two domains had no effect on viral IRES activity, likely due to the unalterable hnRNP A1 localization. As expected, 2A^C110A^ had no effect on the viral IRES activity, consistent with our previous results (Figure 2C).

To further investigate the effect of Hsp27 domain truncations on EV‐A71 replication, we measured the intracellular EV‐A71 RNA level 9 h post infection. The truncation of WDPF domain dramatically decreased the viral RNA by 57% (Figure 2D). In Hsp27∆ACD expressing cells, viral RNA was significantly reduced by 39% (Figure 2D). However, Hsp27∆Flex only mildly reduced the viral RNA level. In cells expressing Hsp27 mutants with two‐domain deletions, the viral RNA level was decreased due to the absence of WDPF or ACD domains (Figure 2D), indicating the critical role of WDPF and ACD domains in IRES‐mediated translation during EV‐A71 infection.

### 2.4 Blocking Hsp27/hnRNP A1 translocation by WDPF peptide

**FIGURE 3:**
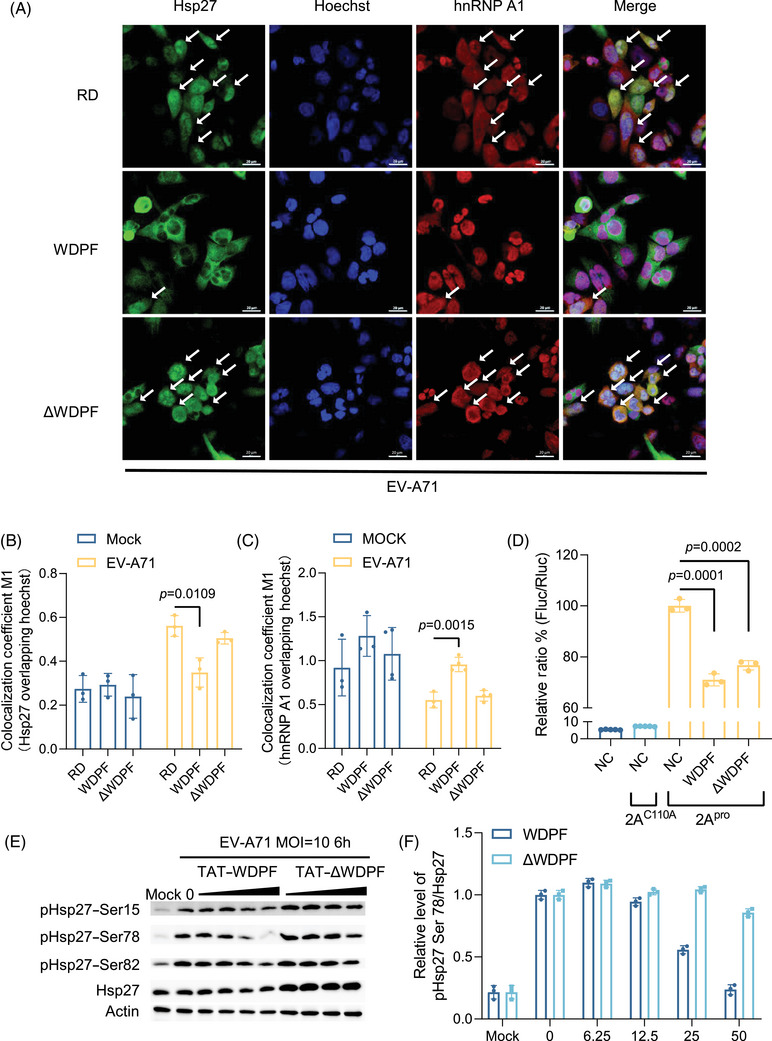
Blockage of Hsp27/hnRNP A1 relocalization and viral IRES activity by WDPF via inhibition of Hsp27 phosphorylation. (A) RD cells on the coverslips were treated with peptides (WDPF and ΔWDPF) at the concentration of 25 µΜ for 2 h, and then infected with EV‐A71 at the MOI of 40 for 6 h. The cells were fixed and stained with anti‐Hsp27 (green) and anti‐hnRNP A1 (red), followed by Alexa Fluor 488‐conjugated anti‐rabbit antibody and Alexa Fluor 594‐conjugated anti‐mouse antibody. The nuclei were stained with Hoechst (Blue). The images were captured by Nikon A1HD25 Confocal Microscope. For Hsp27, cells with nuclear translocation were marked by white arrows. For hnRNP A1, cells with cytosol translocation were marked by white arrows. The scale bar is 20 µM. (B and C) The JACoP‐plugin of the extended ImageJ version Fiji was used to compute the M1 colocalization coefficient (the fraction of Hsp27 in Hoechst or the fraction of hnRNP A1 in Hoechst). (D) HEK 293T cells were pretreated with peptides WDPF or ΔWDPF at the concentration of 25 µΜ for 2 h and then cotransfected with Hsp27–WT plasmid (800 ng), pIRES reporter plasmid (200 ng) and 2A^pro^‐expressing plasmid or 2A^C110A^‐expressing plasmid (200 ng) for 24 h, then the luciferase activity was measured. (E) RD cells were pretreated with peptides WDPF or ΔWDPF for 2 h and infected with EV‐A71 at the MOI of 10 for 6 h. Cell lysates were collected for western blot assay. Hsp27 phosphorylation on S15, S78, and S82 were detected by specific antibodies. β‐Actin were detected as internal control. (F) Densitometric analysis of Figure 3E. Statistical analyses were carried out using Student's *t*‐test. Data are expressed as mean ± SD.

We postulated that targeting WDPF domain may block Hsp27/hnRNP A1 translocation and thereby inhibit viral propagation. We then designed a peptide, named WDPF, which comprises residues 16–27 of Hsp27 and a TAT‐tag sequence for membrane penetration (Figure S3A). It was hypothesized that WDPF would be able to abrogate the WPDF function of endogenous Hsp27, while the controlled peptide, ΔWDPF, without the consensus residues, would have less effect on Hsp27 function. The CC_50_ (50% cytotoxic concentration) of the WDPF and ΔWDPF peptides were 122 µM, and over 600 µM, respectively (Figure S3B,C). After pretreatment with the peptides for 2 h, RD cells were infected with or without EV‐A71 at the MOI of 40 for 6 h to investigate the localization of Hsp27 and hnRNP A1. Without EV‐A71 infection, the localization of Hsp27 and hnRNP A1 was not affected by peptide treatment (Figure S4A). In RD cells, EV‐A71 infection induced the nuclear translocation of Hsp27 and the cytosol translocation of hnRNP A1 (Figure 3A). Notably, WDPF significantly inhibited the translocation of both Hsp27 and hnRNP A1, while peptide ΔWDPF failed to do so (Figure 3A–C).

The effect of the WDPF and ΔWDPF peptides on 2A^pro^‐induced Hsp27/hnRNP A1 translocation was also examined. After 2‐h pretreatment with the peptides, RD cells were transfected with 2A^pro^ or 2A^C110A^ for 24 h. As expected, 2A^pro^ successfully induced Hsp27/hnRNP A1 translocation (Figure S3D), while 2A^C110A^ failed to do so (Figure S4B). Consistent with EV‐A7‐induced translocation, WDPF also blocked the 2A^pro^‐induced translocation of Hsp27 and hnRNP A1 to the basal level, as shown by the quantitative results (Figure S3E,F). In contrast, peptide ΔWDPF treatment did not impede the induction of both Hsp27 nuclear translocation and hnRNP A1 cytosol translocation. Taken together, WDPF effectively inhibited Hsp27/hnRNP A1 translocation which induced by either EV‐A71 infection or 2A^pro^ transfection.

### 2.5 Inhibitory effects of WDPF peptide on S78 phosphorylation of Hsp27 and viral IRES activity

We have previously demonstrated that the phosphorylation of Hsp27 at S78 plays an essential role in the Hsp27/hnRNP A1 translocation and viral IRES‐dependent translation.^31^ Clouser et al.^41^ reported that the WDPF domain and S15 are in the vicinity of S78 and S82 in space. We hypothesized that the inhibitory effect of WDPF on Hsp27/hnRNP A1 translocation may be associated with S78 phosphorylation. To examine this, we investigated the phosphorylation status of Hsp27 at each phosphorylation site upon EV‐A71 infection. Interestingly, WDPF, but not ΔWDPF, inhibited the phosphorylation of Hsp27 dramatically at S78 in a dose‐dependent manner (Figure 3E). Quantification of the data showed that the phosphorylation level at S78 was reduced by 75% with 50 µM‐WDPF treatment (Figure 3F). The phosphorylation level of S15 and S82 were only mildly inhibited by 50 µM‐WDPF treatment (Figure 3E). Furthermore, we examined the effect of peptides on the 2A^pro^‐induced IRES activity. WDPF decreased the 2A^pro^‐induced IRES activity by around 30%, which is a more substantial reduction compared with the ΔWDPF peptide (Figure 3D). Taken together, WDPF specifically inhibited S78 phosphorylation of Hsp27, which in turn hampered the Hsp27/hnRNP A1 translocation and viral IRES activity.

### 2.6 Inhibitory effects of WDPF peptide on EV‐A71 propagation

**FIGURE 4:**
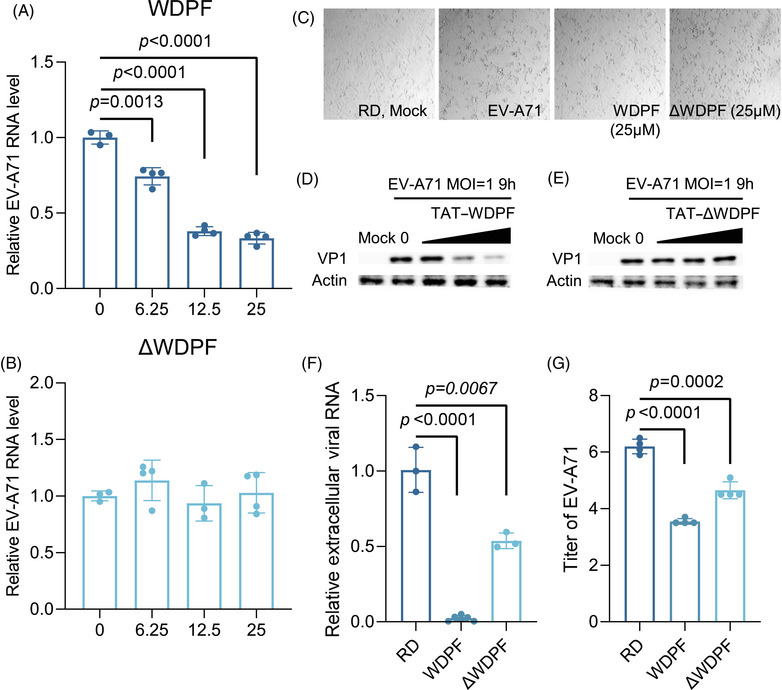
Inhibition of EV‐A71 replication and propagation by WDPF. (A and B) RD cells were treated with peptides WDPF or ΔWDPF and then infected with EV‐A71 at the MOI of 1 for 9 h. Intracellular viral RNA was extracted and quantified by qPCR. The EC_50_ of the WDPF peptide is 7.96 µM. (C) RD cells were treated with peptides and then infected with EV‐A71 at the MOI of 1 for 12 h. The EV‐A71‐induced CPE was captured. (D and E) EV‐A71 VP1 were detected. (F) RD cells were treated with peptides WDPF or ΔWDPF and then infected with EV‐A71 at the MOI of 1 for 12 h. Extracellular viral RNA was extracted and quantified by qPCR. (G) Viral titration was calculated by TCID_50_. Statistical analyses were carried out using Student's *t*‐test. Data are expressed as mean ± SD.

To further investigate the potential antiviral effects of WDPF on EV‐A71 infection, we conducted a cytopathic effects (CPE) assay. In the control group, EV‐A71 infection induced severe CPE at the MOI of 1 for 12 h. We found that the WDPF peptide, but not ΔWDPF, effectively protected the cells from EV‐A71‐induced CPE (Figure 4C). Western blot experiments revealed that the WDPF peptide dramatically reduced the VP1 protein level of EV‐A71 in a dose‐dependent manner, while peptide ΔWDPF had no effect on VP1 level (Figure 4D,E). Consistently, the WDPF peptide but not ΔWDPF, reduced the viral RNA level in a dose‐dependent manner (Figure 4A,B). Specifically, the WDPF treatment decreased the viral RNA level by 65% at 25 µM and reduced the extracellular viral RNA level by 98% (Figure 4F). More strikingly, WDPF treatment achieved over a 450‐fold reduction in virus yield, while peptide ΔWDPF only decreased the viral titer by 40 folds (Figure 4G). Taken together, WDPF displayed a potent antiviral effect against EV‐A71 infection with high specificity.

To explore the potential effects of WDPF on viral entry, RD cells were pretreated with the WDPF peptide or ΔWDPF for 2 h and then infected with EV‐A71 for 1 h (Figure S5A). The cytosolic viral RNA levels were not affected by the peptide treatment, indicating that WDPF and ΔWDPF peptides have no effect on the entry of EV‐A71 (Figure S5B,C).

Furthermore, RD cells were infected with EV‐A71 for 1 h and then treated with the WDPF peptide or ΔWDPF to assess the effect on postentry viral replication (Figure S6A). Importantly, the cytosolic viral RNA was reduced in a dose‐dependent manner by WDPF, but not by ΔWDPF (Figure S6B,C). The half‐maximal effective concentration (EC_50_) of the WDPF peptide was 29.11 µM (Figure S6D). The viral protein VP1 level also showed a dose‐dependent reduction by the WDPF peptide, but not by ΔWDPF (Figure S6E). On the contrary, a peptide NT‐WDPF without the TAT sequence has no effect on EV‐A71 RNA level or protein level (Figure S7A,B,D), indicating that the WDPF peptide functions intracellularly without affecting viral entry.

Furthermore, Hsp27–WT or Hsp27–S78A was expressed in Hsp27 KO cells. The peptide WDPF significantly decreased EV‐A71 RNA level and protein level in Hsp27–WT‐expressing cells (Figure S8A,C). However, the WDPF peptide failed to reduce the EV‐A71 RNA level or protein level in neither Hsp27–S78A‐expressing cells nor Hsp27–KO cells (Figure S8A,B,D), emphasizing the importance of S78 phosphorylation for the antiviral ability of the WDPF peptide.

These results confirmed that the WDPF peptide exerts its antiviral effects after viral entry, hindering the Hsp27/hnRNP A1 translocation through blockage of Hsp27 S78 phosphorylation, thereby suppressing EV‐A71 replication and translation. Targeting WDPF domain shows strong antiviral potent for the development of anti‐EV‐A71 drugs.

### 2.7 Potent antiviral activity of WDPF peptide against both RNA virus HCoV‐OC43 and DNA virus HSV‐1 infection

**FIGURE 5:**
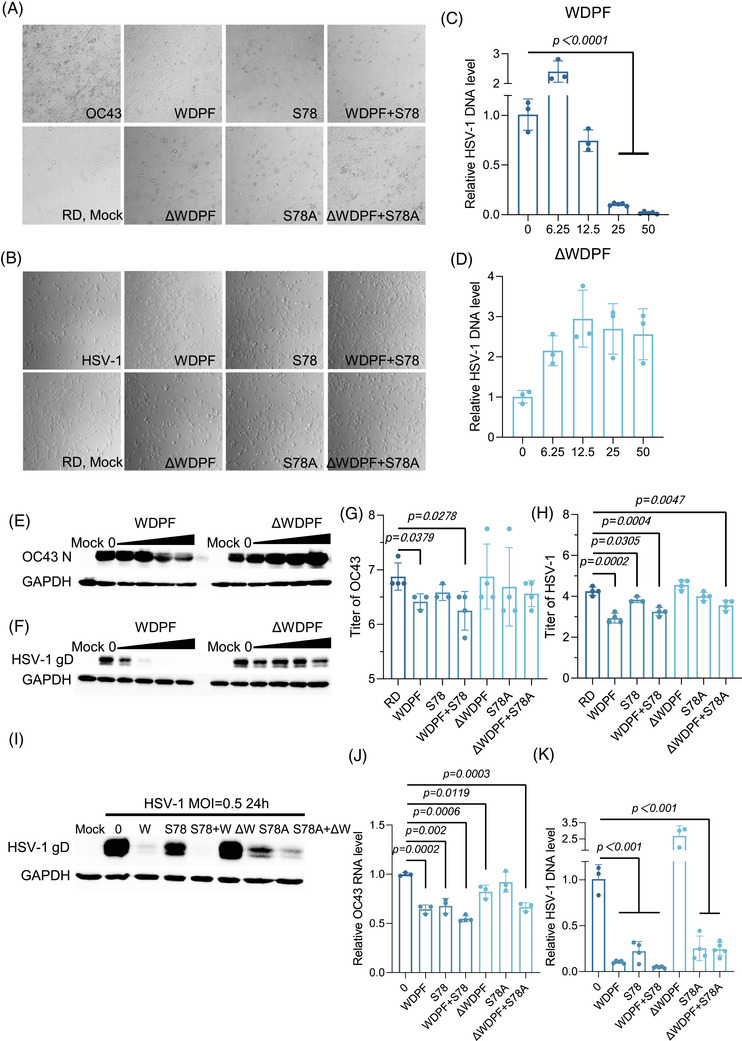
Inhibition of HCoV‐OC43 and HSV‐1 replication and propagation by WDPF. (A and B) RD cells were treated with peptides and then infected with HCoV‐OC43 at the MOI of 1 for 48 h or with HSV‐1 at the MOI of 0.5 for 24 h. The virus‐induced CPE was captured. (C and D) Intracellular viral DNA was extracted and quantified by qPCR. The EC_50_ of the WDPF peptide is 15.52 µM. (E and F) HCoV‐OC43 N protein or HSV‐1 gD protein were detected. (G and H) Viral titration was calculated by TCID_50_. (I) RD cells were pretreated with peptides at the concentration of 25 µM. For the combined peptide treatment, the concentration of each peptide is 25 µM. Then, the cells were infected with virus. HSV‐1 gD protein were detected. (J) Intracellular viral RNA was extracted and quantified by qPCR. (K) Intracellular viral DNA was collected and quantified by qPCR. Statistical analyses were carried out using Student's *t*‐test. Data are expressed as mean ± SD.

Considering that Hsp27 is associated with the life cycle of multiple viruses, we investigated whether the WDPF peptide also suppresses infections by other types of viruses. Hsp27 was reported to be upregulated in SARS‐CoV2‐infected patients.^43^ We found that hnRNP A1 knockdown dramatically reduced the RNA level of an enveloped RNA virus, human coronavirus HCoV‐OC43 (Figure S9A). Then, we further tested the effect of the WDPF peptide on the infection of HCoV‐OC43. Using a CPE assay in RD cells at the MOI of 1 for 48 h, we found that the WDPF peptide successfully protected CPE from HCoV‐OC43 infection, while peptide ΔWDPF had only mild protection (Figure 5A). The WDPF peptide decreased the HCoV‐OC43 RNA level by near 70%, while peptide ΔWDPF only mildly reduced the RNA level by about 10% at the concentration of 25 µM (Figure S10D,E). The nucleocapsid (N) protein level of HCoV‐OC43 was effectively reduced by the WDPF peptide, while peptide ΔWDPF had no detectable effect on the viral protein level (Figure 5E). In terms of viral propagation, the WDPF peptide decreased the viral titer by 65%, while peptide ΔWDPF showed no effect on virus yield (Figure 5G).

The depletion of Hsp27 has been verified to reduce the herpes simplex virus 1 (HSV‐1) replication.^44^ We also found that hnRNP A1 knockdown markedly decreased the DNA level of HSV‐1 (Figure S9B). HSV‐1, an enveloped double‐strained DNA virus, infects humans and causes painful blisters or ulcers and is associated with an increased risk of Alzheimer's disease, amyotrophic lateral sclerosis, and other central nervous system disorder.^45^, ^46^ We further examined the antiviral effects of the WDPF peptide on HSV‐1 infection. The WDPF peptide markedly inhibited HSV‐1‐induced CPE, while ΔWDPF had no inhibitory effect (Figure 5B). The WDPF peptide effectively reduced the intracellular HSV‐1 DNA level in a dose‐dependent manner, with a 90% reduction at 25 µM (Figure 5C), while ΔWDPF failed to decrease the viral DNA level (Figure 5D). The WDPF peptide, but not peptide ΔWDPF, largely decreased HSV‐1 glycoprotein D (gD) protein level in a dose‐dependent manner (Figure 5F) and reduced the viral titer by more than 20 folds (Figure 5H).

Hepatitis type B virus (HBV) is a DNA virus undergoing reverse‐transcription in the life cycle and a main cause of viral hepatitis, liver cirrhosis and hepatocellular carcinoma.^47^, ^48^, ^49^ It was reported that high expression of Hsp27 correlates with the prognosis of the patients with HBV‐associated hepatocellular carcinoma.^50^ It is a great interest for us to know if the WDPF peptide inhibits HBV replication. We showed that the peptide WDPF decreased the core promoter activity by over 35% shown by a luciferase reporter assay, while peptide ΔWDPF has little effects on the luciferase reporter activity (Figure S11A). These results indicate that the WDPF peptide inhibits the transcription of HBV pregenomic RNA and corresponding viral protein expression. The WDPF peptide dramatically decreased the HBV DNA level by 82%, however peptide ΔWDPF had no effect on the DNA level (Figure S11B). Thus, the WDPF peptide suppresses the replication and transcription of HBV.

Given that the WDPF peptide can inhibit S78 phosphorylation of Hsp27 in a dose‐dependent manner, it could function through the same mechanism as the peptide S78.^31^ We also assessed the effects of S78 and the control peptide S78A on HCoV‐OC43 and HSV‐1 infection. At 25 µM, S78 and S78A decreased the HCoV‐OC43 RNA level by 55% and 46%, respectively (Figure S10F,G). For HCoV‐OC43 reproduction, S78 effectively reduced the viral titer by 65%, while S78A mildly decreased the viral titer by 35% (Figure 5G). For HSV‐1, the gD protein and DNA levels were reduced by S78 and S78A in a dose‐dependent manner (Figure S10A–C). S78 and S78A decreased the HSV‐1 titer by 68% and 44%, respectively (Figure 5H). However, the inhibitory effect of S78 was milder than that of the WDPF peptide. For HBV, S78 and S78A have no dramatic effect on both the core promoter activity and DNA level (Figure S11), indicating that the WDPF peptide may not inhibit HBV infection through the suppression of S78 phosphorylation of Hsp27.

To investigate the combined effect of the peptides, RD cells were treated with 25 µM of peptides WDPF and S78 before virus infection. As shown in Figure 5A,B, the WDPF and S78 combination effectively protected CPE from HCoV‐OC43 and HSV‐1 infection, while the ΔWDPF and S78A combination did not show dramatic inhibition on the virus‐induced CPE. The WDPF and S78 combination also further suppressed HSV‐1 protein (Figure 5I). For the HCoV‐OC43 RNA level and viral titer, the WDPF and S78 combination had a similar effect to the WDPF or S78 treatment alone (Figure 5G,J), indicating that these two peptides suppress the HCoV‐OC43 infection through the same mechanism. However, in the cases of HSV‐1 and HBV infections, the antiviral activity of WDPF and S78 combination was similar to that of the WDPF peptide (Figures 5H,K and S11), no synergistic effect was observed.

Additionally, we investigated the effect of the peptides on the interferon‐sensitive response element (ISRE) activity. HEK293T cells were transfected with Hsp27, pISRE (Fluc‐expressing), and pRF (Rluc‐expressing) luciferase reporter plasmids and treated with peptides. The normalized ratio of Fluc to Rluc, which represented the relative ISRE activity, was not affected by the peptide treatments after recombinant IFNα stimulation (Figure S12). Therefore, the inhibitory effect of the peptides on virus infection is not through the induction of the interferon response.

### 2.8 Additional antiviral activity of WDPF peptide and Sorafenib combination

Sorafenib, an inhibitor of multiple kinases including serine, threonine, and tyrosine kinases, is an FDA‐approved drug for treating primary kidney cancer and liver cancer.^51^, ^52^ It was reported that Sorafenib inhibits EV‐A71 replication through the regulation of viral IRES‐dependent protein translation.^53^ We investigated whether the peptide could achieve additional antiviral effects when combined with Sorafenib treatment.

**FIGURE 6:**
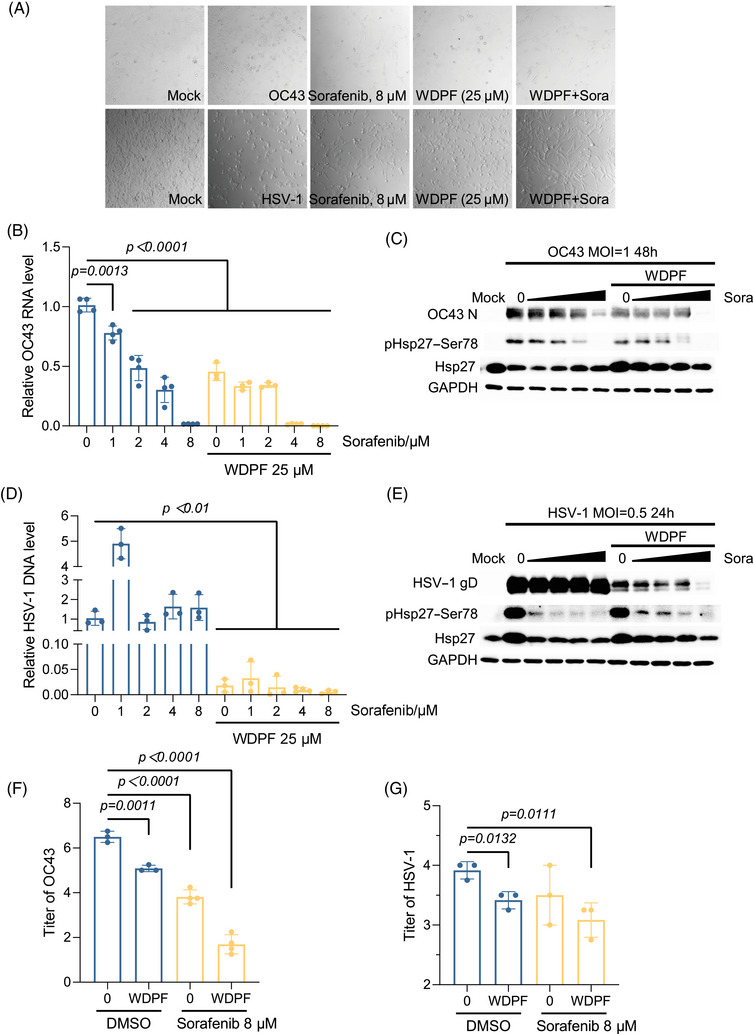
Inhibition of HCoV‐OC43 and HSV‐1 replication and propagation by combined treatment of Sorafenib and WDPF. (A) RD cells were treated with Sorafenib and WDPF at indicated concentration and then infected with HCoV‐OC43 (MOI = 1, 48 h) or HSV‐1 (MOI = 0.5, 24 h). The virus‐induced CPE was captured. (B) Intracellular viral RNA was extracted and quantified by qPCR. (C) The protein levels of HCoV‐OC43 N protein, pHsp27–S78, and Hsp27 were detected. (D) Intracellular viral DNA was extracted and quantified by qPCR. (E) The protein levels of HSV‐1 gD protein, pHsp27–Ser78, and Hsp27 were detected. (F and G) Viral titration was calculated by TCID_50_. Statistical analyses were carried out using Student's *t*‐test. Data are expressed as mean ± SD.

The CPE assay demonstrated that Sorafenib treatment largely protected the cells from HCoV‐OC43 infection at 8 µM, but only mildly inhibited HSV‐1‐induced CPE (Figure 6A). Sorafenib decreased the HCoV‐OC43 RNA level in a dose‐dependent manner, with a 98% reduction at a concentration of 8 µM. The results showed that the WDPF peptide enhanced Sorafenib's antiviral effects, as the same level of RNA reduction was achieved at only 4 µM of Sorafenib, indicating additional antiviral effect (Figure 6B). Consistent with the viral RNA reduction, the N protein level of HCoV‐OC43 decreased to an undetectable level by western blot assay when Sorafenib was used in combination with 25 µM of the WDPF peptide (Figure 6C). The WDPF and Sorafenib individually reduced the HCoV‐OC43 titer by 1.4 and 2.7 logs, respectively, while the combined treatment of WDPF and Sorafenib decreased the titer by 4.8 logs (Figure 6F). Furthermore, the WDPF combined with Sorafenib completely blocked S78 phosphorylation of Hsp27, further demonstrating its importance in virus propagation (Figure 6C).

In the case of the DNA virus HSV‐1, Sorafenib failed to suppress viral DNA replication and protein synthesis, although it potently inhibited the phosphorylation at S78 of Hsp27 (Figure 6D,E). Notably, the WDPF alone potently reduced the DNA level by 98%. Combined with 8 µM of Sorafenib treatment, the DNA level of HSV‐1 was further decreased by 70% (Figure 6D); and interestingly, the viral gD protein level significantly decreased in a Sorafenib dose‐dependent manner (Figure 6E). The combined treatment decreased the titer by 85%, while the WDPF peptide and Sorafenib reduced it by 68 and 62%, respectively (Figure 6G). In summary, the combined treatment of the WDPF and Sorafenib achieves additional inhibitory effects on both HCoV‐OC43 and HSV‐1 infection.

## 3 DISCUSSION

Hsp27 functions through its dynamic structure regulated by oligomerization and oligomer dissociation.^54^ Hsp27 consists of a less conserved N‐terminal region (NTR) containing a WDPF domain, a highly conserved ACD domain, and a flexible C‐terminal domain. The ACD domain forms two β‐sheets with six β‐strands, providing a β4/β8 groove and a dimer interface groove for oligomerization and protein interaction.^55^, ^56^ The flexible C‐terminal domain is responsible for Hsp27 oligomers formation and solubility.^38^ The IXI motif near the C‐terminus interacts with the β4/β8 groove of the ACD domain.^57^, ^58^ The N‐terminal WDPF domain is involved both in the oligomer formation and the chaperone activity.^59^ Clouser et al.^41^ have reported that four distinct regions of NTR interact with the ACD domain, including the distal region (1‐13) with the β4/β8 groove, the aromatic region (12–27) with loops L3/4 and L5/6, the conserved region (25–37), and the boundary region (74–91) with the dimer interfaced groove. The complicated composition contributes to the dynamic Hsp27 oligomer size and its diverse functions. Furthermore, the aromatic region (12–27), including WDPF motif and serine 15, is in close spatial proximity to S78 and S82. Thus, all the three phosphorylation sites are located near the negatively charged ACD region, making them susceptible to phosphorylation‐induced changes.^41^ Interestingly, Hsp27 phosphorylation in response to various stresses is responsible for the tunability of Hsp27 oligomers.^39^ Previously, we reported that Hsp27 is hijacked by EV‐A71 to facilitate Hsp27/hnRNP A1 translocation and EV‐A71 replication through phosphorylation of S78 of Hsp27.^30^, ^31^ Thus, we hypothesized that Hsp27 domains may play critical roles in promoting EV‐A71 infection, in conjunction with Hsp27 phosphorylation.

In this study, the deletion of ACD domain led to low expression level of Hsp27ΔACD, Hsp27ΔACD/WDPF, and Hsp27ΔACD/Flex, indicating the essential role of ACD domain for Hsp27 expression as a highly conserved core domain. The low expression of these mutants could result in the disturbance of Hsp27 function and probably the absence of Hsp27‐mediated mechanisms. Under this intricated condition, little expression of the Hsp27 mutants has the similar effect with knockout of Hsp27. Thus, the effect of the ACD domain remain complicated. The flexible C‐terminal domain deletion did not significantly affect the hnRNP A1‐mediated EV‐A71 translation. Importantly, the deletion of WDPF domain induced Hsp27 to localize in both cytoplasm and nucleus. However, upon EV‐A71 infection, the nuclear translocation of Hsp27–ΔWDPF failed to promote cytosolic relocalization of hnRNP A1 (Figures 1 and 2).

The WDPF peptide, developed to competitively inhibit the endogenous Hsp27 WDPF function, did not affect the subcellular localization of endogenous Hsp27 (Figure 3), suggesting that the deletion of the WDPF domain may generate an unknown NLS or alter the Hsp27 conformation to facilitate Hsp27 nuclear import. However, the WDPF peptide dramatically suppressed Hsp27 phosphorylation at S78, accompanied by the blockage of hnRNP A1 relocalization and reduction of viral IRES activity. The WDPF peptide also strongly protected RD cells from the EV‐A71‐induced CPE, and dramatically decreased viral replication and translation. The viral titer was decreased by over 450 folds in cells treated with the WDPF peptide (Figure 4). In contrast, peptide ΔWDPF, with a shorter region of domain WDPF, failed to suppress Hsp27 phosphorylation at any serine site, hamper hnRNP A1 translocation, or inhibit viral IRES‐dependent translation and viral reproduction.

HSV‐1 is a double‐stranded DNA virus. It was reported that HSV‐1 induces Hsp27 phosphorylation and translocation into nucleus.^44^ Interestingly, knockdown of hnRNP A1 reduced the DNA level of HSV‐1. Then, we investigated the effect of WDPF peptide on HSV‐1 infection. The WDPF peptide was also able to largely suppress the replication and reproduction of HSV‐1 (Figure 5). Furthermore, the kinase inhibitor Sorafenib treatment was demonstrated to enhance the inhibition of HSV‐1 replication and propagation by the WDPF treatment (Figure 6). Although Sorafenib can also inhibit the S78 phosphorylation of Hsp27 (Figure 6E), Sorafenib alone failed to decrease the viral replication and translation of HSV‐1. These results suggest that the reduced S78 phosphorylation may be a partial reason for the inhibitory effect of WDPF on HSV‐1 infection. The WDPF peptide may disrupt additional Hsp27‐dependent processes, such as its chaperone function, viral entry and trafficking, host stress response, and impact on apoptosis and cell survival pathways, all of which could impair viral replication and spread.^2^, ^60^, ^61^

It is also amusing that the peptide WPDF also potently inhibits HBV replication, as HBV infection largely contributes to liver disease related death in Asia and Africa. The WDPF peptide significantly decreased both HBV replication and transcription, while the ΔWDPF peptide had no effect (Figure S11). To our surprise, the S78 peptide failed to reduce the HBV replication, indicating that S78 phosphorylation of Hsp27 is not associated with HBV infection. Similar to HSV‐1, the WDPF peptide suppresses HBV replication through other mechanisms.

Additionally, knockdown of hnRNP A1 also reduced the RNA level of HCoV‐OC43. WDPF peptide significantly decreased the viral propagation of HCoV‐OC43 (Figure 5). The combined treatment with the WDPF peptide and Sorafenib had an additional antiviral effect on HCoV‐OC43 infection (Figure 6). These results demonstrate an effective broad‐spectrum antiviral peptide. The combination of new peptide and approved drug further leads to the increased potency and therapeutic impact.

Although the crucial domain and phosphorylation site of Hsp27 involved in the induction of hnRNP A1 redistribution have been identified in this study, the detailed mechanisms underlying this process remain elusive. Previous studies have reported that the poliovirus and rhinovirus 2A^pro^ can cleave the nuclear pore glycoproteins such as NUP62, NUP98, and NUP153, thereby disrupting the nucleocytoplasmic dynamic.^62^, ^63^, ^64^, ^65^ This cleavage can lead to significant alterations in the transport of proteins and RNA between the nucleus and cytoplasm. The rhinovirus 3C protease was shown to cleave Nup153 and induce the redistribution of the essential splicing factors SC35 and nucleolin.^66^ Interestingly, HIV‐1 was reported to alter the abundance and localization of NUP62, thereby blocking the nuclear import pathway that leads to the exported hnRNP A1 accumulates in the cytoplasm.^67^ These studies highlighted that viruses regulate the nuclear transport pathway to benefit their life cycle in the host cells. As an enterovirus, it is plausible that EV‐A71 2A^pro^ could cleave the nuclear pore glycoproteins, resulting in the cytoplasmic accumulation of hnRNP A1. Alternatively, 2A^pro^ may trigger posttranslational modification of hnRNP A1 with the help of Hsp27 to promote hnRNP A1 nuclear export. By dissecting these detailed pathways, we may uncover multiple innovative strategies for developing effective antiviral therapies to mitigate the impact of EV‐A71 and related viral infections.

In summary, Hsp27ΔWDPF lost the normal Hsp27–WT pattern and localized in the nucleus without any stimuli. At the same time, Hsp27ΔWDPF failed to induce hnRNP A1 nuclear translocation upon EV‐A71 infection, leading to further reduction in IRES activity and viral replication. The WDPF peptide was developed to inhibit EV‐A71‐induced hnRNP A1 translocation and viral IRES activity, while peptide ΔWDPF failed to do so. Importantly, the WDPF peptide dramatically suppressed S78 phosphorylation of Hsp27 upon EV‐A71 infection, strongly inhibiting viral replication and propagation. Furthermore, when combined with the approved anticancer drug Sorafenib, the WDPF peptide had additional inhibitory effect on HCoV‐OC43 and HSV‐1 infection, offering a new avenue for the development of broad‐spectrum antiviral therapies.

## 4 MATERIALS AND METHODS

### 4.1 Cells and virus

Human embryonic kidney 293T (HEK293T) cells and rhabdomyosarcoma (RD) cells (ATCC ^#^CCL‐136) were cultured in Dulbecco's modified Eagle's medium with 10% fetal bovine serum, 100 U/mL of penicillin, and 100 µg/mL of streptomycin. Hsp27 knockout RD cells (Hsp27–KO RD cells) were previously generated using the CRISPR/Cas 9 system and maintained in the lab.^30^ The targeting single‐guide RNA sequence was 5′‐GCAUAGCCGCCUCUUCGACC‐3′. All the cells used in this study have undergone the authentication by STR analysis and mycoplasma test. EV‐A71 was obtained from the Shenzhen Center for Disease Control and Prevention (SHZH98 strain, GenBank accession number AF302996). HSV‐1 (HSV‐1, VR‐1493™) and human coronavirus OC43 (HCoV‐OC43, VR‐1558™) were purchased from ATCC. All viruses were propagated, aliquoted, and stored at −80°C until further use, as described previously.^68^

### 4.2 Plasmids

The EV‐A71 IRES reporter plasmid, pcDNA4/HisMax B‐2A^pro^, and pcDNA4/HisMax B‐2A^C110A^ were generated as previously described.^69^, ^70^ The protease‐inactive mutation of 2A^pro^ (Cys^110^ to Ala^110^, 2A^C110A^) was generated by site‐directed mutagenesis with a one‐step mutagenesis kit (Invitrogen). To construct EV‐A71 IRES reporter plasmid, the Rluc gene was inserted into pcDNA4/HisMax B between *Bam*H I and *Eco*R V sites first, then the amplified IRES‐Fluc encoding sequence was inserted downstream of the Rluc using *Eco*R V and *Xba*r I. The primers used in amplification are available upon request. The pcDNA4/HisMax B–Hsp27 and pcDNA4/HisMax B–Hsp27–S78A were described previously.^31^ The HBV (subtype adr) genomic clone pHBV‐adr was a gift from Professor Yuan Wang, Shanghai Institute of Biochemistry, Academia Sinica.^71^ The 1.3xHBV‐Luciferase plasmid to monitor HBV transcription was purchased from Addgene (#61549; Addgene, USA). Luciferase was inserted within Core ORF and regulated by EnhII–Core promoter.

### 4.3 Lentivirus package

Lentiviral vectors were prepared as described previously.^72^ Briefly, the plasmids (pLV–Hsp27–EGFP–Flag, pLV–Hsp27–ΔACD–EGFP–Flag, pLV–Hsp27–ΔWDPF–EGFP–Flag, pLV–Hsp27–ΔFLEX–EGFP–Flag, pLV–Hsp27–ΔACD/WDPF–EGFP–Flag, pLV–Hsp27–ΔACD/FLEX–EGFP–Flag, pLV–Hsp27–ΔWDPF/FLEX–EGFP–Flag, and pLV–hnRNPA1–mcherry–Flag) were constructed by Inovogen Tech. Co. Then, these plasmids were cotransfected with the psPAX2 (packaging) and pMD2.G (envelope) plasmids into HEK293T cells using polyethylenimine as the transfection reagent. The ratio of the plasmids used was psPAX2:pMD2.G:lentiviral transfer plasmid = 6:3:9 µg. The culture medium was replaced 2–4 h after transfection. After 96 h, the supernatant containing the lentiviral particles was collected, aliquoted, and stored at −80°C.

### 4.4 Immunofluorescence assay

We followed a previously reported protocol to study the distribution of hnRNP A1.^30^ In brief, cells cultured on coverslips were infected with EV‐A71 at an MOI of 40 for 6 h to ensure a high efficiency of viral infection. After infection, the cells were fixed with 4% paraformaldehyde for 20 min and permeabilized with 0.5% Triton X‐100 for 15 min. The cells were then blocked with 5% bovine serum albumin (BSA) for 2 h. The cells with stable expression of Hsp27–eGFP and hnRNP A1–mCherry were directly stained with Hoechst. The RD cells treated with peptides were incubated with primary antibodies against Hsp27 (GTX101145) and hnRNP A1 (sc‐32301), followed by incubation with Alexa Fluor 488‐conjugated anti‐rabbit and Alexa Fluor 594‐conjugated anti‐mouse secondary antibodies, respectively. After four washes with 0.2% Triton X‐100, the cell nuclei were stained with Hoechst for 5 min. The stained cells were imaged using a Nikon A1HD25 high‐speed and large field‐of‐view confocal microscope. Colocalization analysis was conducted using the JACoP‐plugin in the Fiji (ImageJ) software. The Mander's M1 colocalization coefficient was computed to quantify the colocalization levels of Hsp27 or hnRNP A1 overlapped with Hoechst.

### 4.5 Luciferase assay

The reporter plasmids used in this study were described previously.^69^ HEK 293T cells were seeded in 24‐wells, and then cotransfected with the following plasmids: 2A^pro^ expression plasmid (200 ng), pIRES reporter plasmid (200 ng), and the plasmid expressing wild‐type Hsp27 or its mutants (800 ng). After culturing for 24 h, the transfected cells were lysed using a passive lysis buffer (Promega, USA). The Rluc and Fluc activities in the cell lysates were measured using the dual‐luciferase reporter assay system (Promega) according to the manufacturer's instructions. The luminescence was detected using a Lumat LB9507 bioluminometer.

### 4.6 RNA isolation

Total cellular RNA was isolated using the established protocol described previously.^73^ Briefly, the cells were lysed with TRIzol reagent (Ambion, Life Technologies), and the intracellular RNA was extracted according to the manufacturer's instructions. Alternatively, TRIzol reagent was added into the supernatant solution of the cells (TRIzol:solution = 1:1), and the extracellular RNA was extracted according to the manufacturer's instructions. For cDNA synthesis, 1 µg of the isolated total RNA was used as the input for reverse transcription. The cDNA was synthesized using the PrimeScript™ RT Master Mix (Takara) following the manufacturer's protocol. For DNA virus, total cellular DNA was isolated using the QIAamp DNA Mini and Blood Mini Extraction Kit.

### 4.7 Quantitative RT‐PCR

Quantitative real‐time PCR (RT‐qPCR) was performed using TB Green® Premix Ex Taq™ (Takara) on the Applied Biosystems QuantStudio™ 3 Real‐Time PCR Systems. The thermal cycling conditions for the RT‐qPCR were as follows: initial activation at 95°C for 30 s, followed by 45 cycles of amplification, with each cycle consisting of 95°C for 5 s and 60°C for 30 s. After the amplification cycles, a melting temperature analysis was carried out by slowly increasing the temperature from 60 to 95°C at a rate of 0.1°C/s to verify the specificity of the amplified products. The primer sequences used for the target gene amplification were:

EV‐A71 VP1, F 5′‐CGGACTGTAGGCACCTCGAA‐3′, R 5′‐CGCATTGGGCGAGGTATC‐3′;

GAPDH, F 5′‐GTCTCCTCTGACTTCAACAGCG‐3′, R 5′‐ACCACCCTGTTGCTGTAGCCAA‐3′;

HSV‐1 UL1, F 5′‐GCCAGCGAGACGCTGAT‐3′, R 5′‐ACGCAGGTACTCGTGGTGA‐3′;

HCoV‐OC43 NSP1, F 5′‐TTGTGAGCGATTTGCGTGCG‐3′, R 5′‐ACACGTCCCTGGCTGAAAGC‐3′;

HBV core, F 5′‐AGTGTGGATTCGCACTCCT‐3′, R 5′‐GAGTTCTTCTTCTAGGGGACCTG‐3′.

The messenger RNA (mRNA) expression levels of each target gene were normalized to the mRNA copies of GAPDH in the same samples, as previously described.^30^

### 4.8 Peptide treatment

The peptides used in this study were constructed and purchased from GL Biochem (Shanghai) Ltd. RD cells were seeded into 24‐well plates and cultured for 24 h. The cells were then pretreated with the peptides at the indicated concentrations for 2 h and were either infected with EV‐A71 or transfected with plasmids for further experiments. The amino acids sequences of the peptides were RKKRRQRRR‐AYSRALSRQLSS (TAT‐S78, S78), RKKRRQRRR‐AYSRALARQLSS (TAT‐S78A, S78A), RKKRRQRRR‐WDPFRDWYPHSR (TAT‐WDPF, WDPF), RKKRRQRRR‐DWYPHSR (TAT‐ΔWDPF, ΔWDPF), and WDPFRDWYPHSR (NoTAT‐WDPF, NT‐WDPF).

### 4.9 Western blot assay

The Western blot protocol used in this study has been described previously.^74^ Briefly, the cells were lysed in Nonidet‐P40 (NP‐40) buffer, and the cell lysates were centrifuged. The protein concentration in the cell lysates was determined using the Bradford assay (Bio‐Rad). A total of 20 µg of protein from each sample was resolved on a 12% SDS‐PAGE gel and then transferred onto a polyvinylidene fluoride membrane (GE, MA, USA). After blocking the membrane with 5% BSA for 1 h, the membrane was incubated with primary antibodies (diluted 1:1000) against the following proteins overnight at 4°C: Hsp27 (sc‐13132; Santa Cruz), pHsp27–Ser15 (ab76313; abCam), pHsp27–Ser78 (ADI‐SPA‐523; Enzo), pHsp27–Ser82 (ab155987; abCam), VP1 (GTX132339; GeneTex), Actin (sc‐47778; Santa Cruz)), hnRNP A1 (sc‐32301; Santa Cruz)), OctA–Probe (Flag, sc‐166355; Santa Cruz)), HSV‐1 gD (sc‐21719; Santa Cruz), HCoV‐OC43 N (40643‐T62; Sino), or GAPDH (sc‐32233; Santa Cruz)) overnight in 4°C. After secondary antibodies (HRP‐conjugated Affinipure Goat Anti‐Mouse IgG(H+L), SA00001‐1, and HRP‐conjugated Affinipure Goat Anti‐Rabbit IgG(H+L), SA00001‐2; ProteinTech) incubation for 2 h, the protein band was visualized using a chemiluminescence reagent (PerkinElmer, MA, USA) and imaged using the Bio‐Rad Chemidoc Imaging System. If necessary, the protein bands were quantified by densitometry analysis using ImageJ software.

### 4.10 Virus titration

RD cells were seeded into 96‐well plates and incubated for 24 h. The cells were then infected with 100 µL per well of serially diluted virus supernatant, with each dilution tested in quintuplicate. The serial dilutions of the virus supernatant were prepared as 10‐fold dilutions in the appropriate cell culture medium. After 120 h of incubation postinfection, the 50% tissue culture infectious dose (TCID_50_) was calculated using the Kärber method, as described previously.^69^, ^70^

### 4.11 RNA interference

A small interfering RNA (siRNA) targeting human hnRNP A1 mRNA (si hnRNP A1) was designed (sense primer 5′‐GCUCUUCAUUGGAGGGUUG‐3′) and synthesized by GenePharma (Shanghai, China). Western blot assays were applied to measure the knockdown efficiency of the siRNA. A nonspecific siRNA with no homology to the human genome was used as the negative control.

### 4.12 CCK‐8 assay

RD cells were seeded in 96‐well cell plates at a density of 1 × 104 cells per well. The cells were then placed in a 5% CO_2_ incubator for 24 h. Then, the cells were treated with the peptides at the desired concentrations for 2 days. A total of 90 µL of fresh cell culture medium and 10 µL of CCK‐8 (BS350B; BioSharp) were added to each well of the 96‐well plate, which were incubated at 37°C for 1 h. The absorbance of each well was measured using a microplate reader (Synergy H1; BioTek). The absorbance was recorded at a wavelength of 450 nm.

### 4.13 Statistical analysis

The experimental results were expressed as the mean ± standard deviation (SD) for each experimental group. All statistical analyses were carried out using the GraphPad Prism 8.0 software (GraphPad Inc.). For comparisons between two experimental groups, a two‐tailed Student's *t*‐test was applied. A *p* value less than 0.05 (*p* < 0.05) was considered statistically significant.

## AUTHOR CONTRIBUTIONS

*Conceptualization*: Ming‐Liang He *Methodology*: Mandi Wu and Wei Li *Formal analysis*: Mandi Wu, Wei Li, and Houying Leung *Investigation*: Mandi Wu, Wei Li, Houying Leung, Yiran Wang, Qianya Wan, Peiran Chen, Cien Chen, Yichen Li, Xi Yao, and Ming‐Liang He *Resources*: Mandi Wu, Wei Li, Houying Leung, Yiran Wang, Qianya Wan, Peiran Chen, Cien Chen, Yichen Li, Xi Yao, and Ming‐Liang He *Original draft preparation*: Mandi Wu *Review and editing*: Ming‐Liang He, Mandi Wu, Wei Li, and Yiran Wang *Visualization*: Ming‐Liang He and Mandi Wu *Supervision*: Ming‐Liang He *Project administration*: Ming‐Liang He *Funding acquisition*: Ming‐Liang He. All authors have read and agreed to the published version of the manuscript.

## CONFLICT OF INTEREST STATEMENT

Author Ming‐Liang He is an editorial board member of MedComm. Author Ming‐Liang He was not involved in the journal's review of or decisions related to this manuscript. The other authors declared no conflict of interest.

## ETHICS STATEMENT

Not applicable, as there are no animal/clinical experiments in this study.

## Supporting information



Supporting Information

## Data Availability

The raw data generated from this study are available from the corresponding author M. L. H. on request.
