# Supplementary material for: Targeting WDPF domain of Hsp27 achieves a broad spectrum of antiviral
Source: MedComm (2020). 2025 Feb 26;6(3):e70032. doi: 10.1002/mco2.70032 (PMC11862887; doi:10.1002/mco2.70032)
Supplement: Supplementary file 1 — Supporting Information [file MCO2-6-e70032-s001.pdf]

Targeting WDPF domain of Hsp27 achieves a broad spectrum of antiviral

Mandi Wu [1,#], Wei Li [2,#], Houying Leung [1], Yiran Wang [1], Qianya Wan [1], Peiran Chen [1], Cien Chen [1], Yichen Li [1], Xi Yao[1,*], and Ming-Liang He [1,3,*]

[1] Department of Biomedical Sciences, City University of Hong Kong, Hong Kong Special Administrative Region, China

[2] Weihai Municipal Hospital, Cheeloo College of Medicine, Shandong University, Weihai, Shandong, 264200, China

[3] CityU Shenzhen Research Institute, Nanshan, Shenzhen, China

# Those authors contributed equally

* Correspondence: mlhe7788@gmail.com or minglihe@cityu.edu.hk; xi.yao@cityu.edu.hk

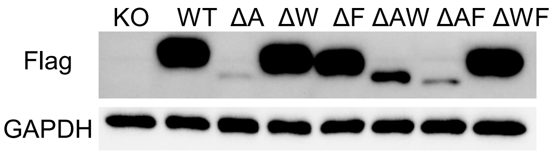

Figure S1 Expression level of Hsp27 WT and mutants

Hsp27 WT and its mutants (Hsp27ΔACD, Hsp27ΔWDPF, Hsp27ΔFLEX, Hsp27ΔACD/ΔWDPF, Hsp27ΔACD/ΔFLEX, Hsp27ΔWDPF/ΔFLEX) were stably expressed in Hsp27-KO RD cells by lentiviral vectors. Cell lysates were collected for western blot assay.

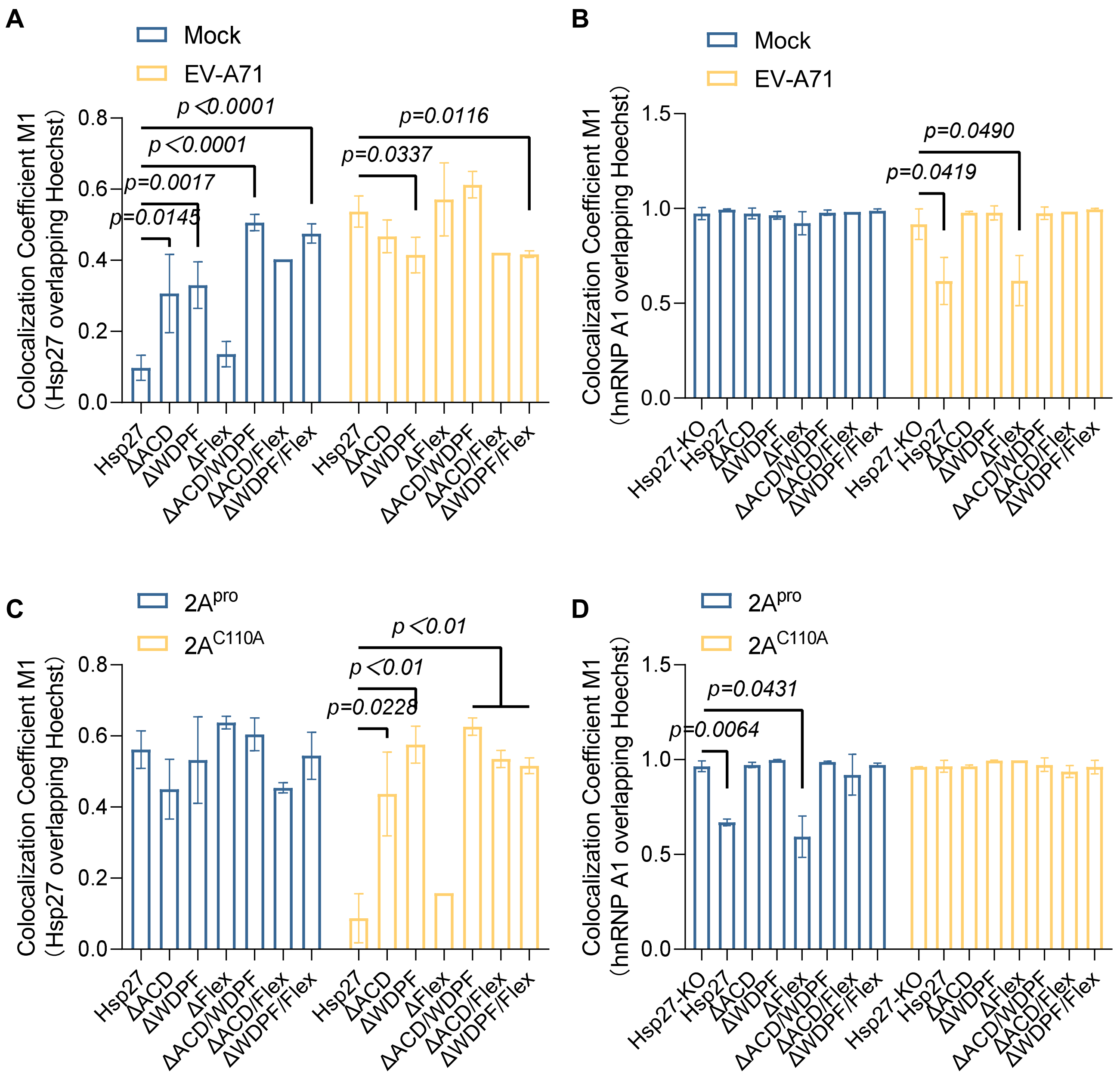

Figure S2 Quantification of the colocalization of Hsp27, hnRNP A1, and Hoechst in Hsp27 truncations-restoring cells with infection of EV-A71 or ectopic expression of $2A^{pro}$ and $2A^{C110A}$

A-D. The JACoP-plugin of the extended ImageJ version Fiji was used to compute the M1 colocalization coefficient (the fraction of Hsp27 in Hoechst or the fraction of hnRNP A1 in Hoechst). The fraction of Hsp27 in Hoechst or the fraction of hnRNP A1 in Hoechst was quantified, as the images were shown in Fig. 1-2. Statistical analyses were carried out using Student’s t-test. Data are expressed as mean ±SD.

A

WDPF
RKKRRQRRR WDPFRDWYPHSR
ΔWDPF
RKKRRQRRR DWYPHSR

B

Peptide	WDPF	ΔWDPF
CC_{50}	122 μM	> 600 μM

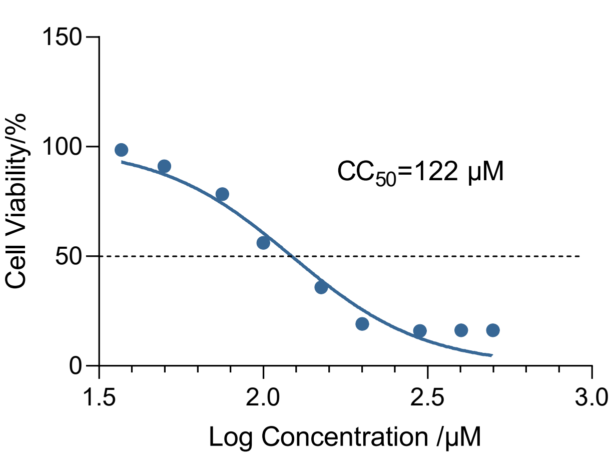

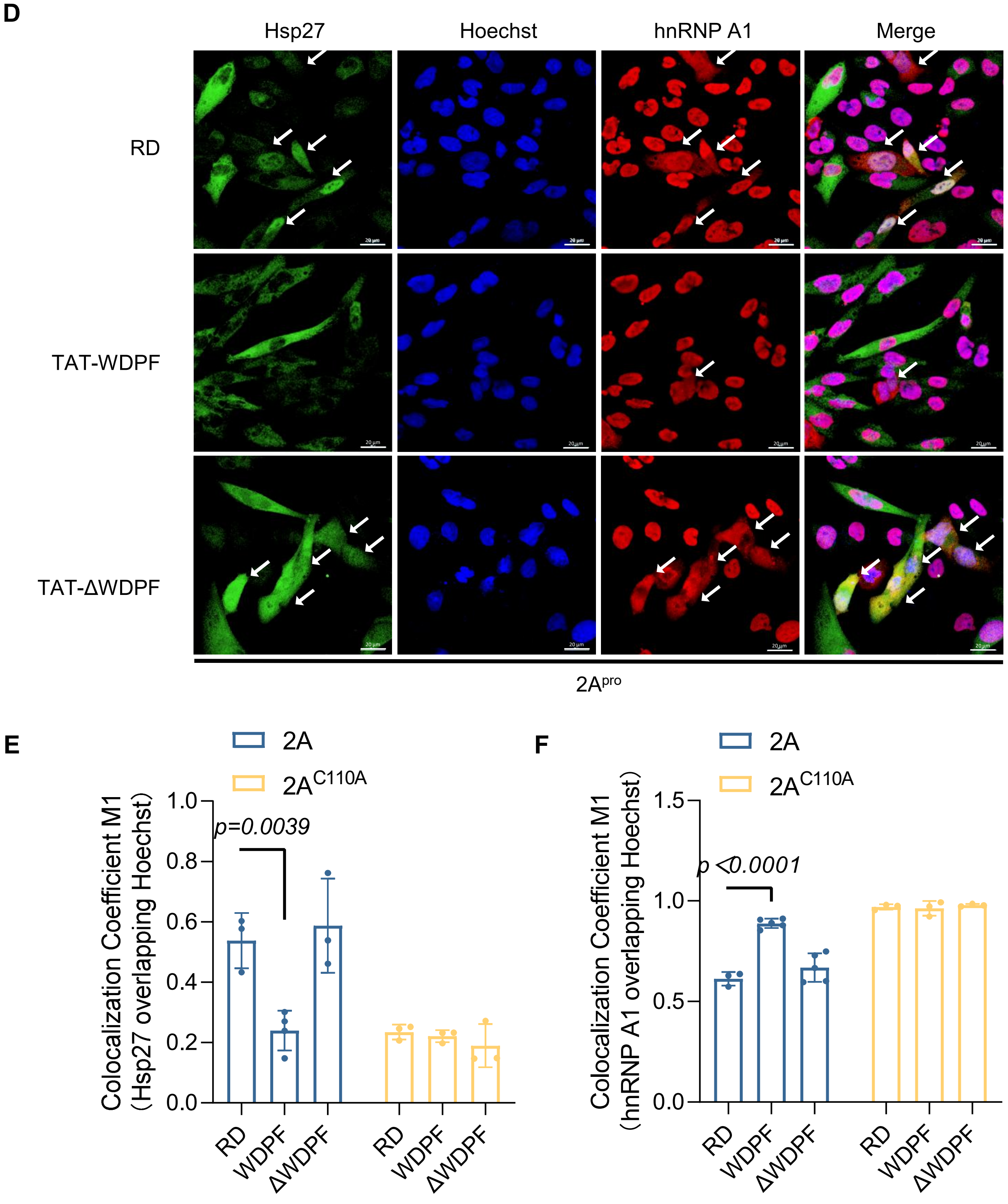

Figure S3 CC_{50} of peptides and blockage of $2A^{pro}$-induced Hsp27/ hnRNP A1 re-localization by peptide WDPF

A. The amino acid sequence of peptide WDPF and ΔWDPF. **B.** RD cells were treated with peptides WDPF or ΔWDPF for 48 hours and CCK-8 assay was used to calculate CC_{50} of the peptides. **C.** The cell viability of peptide WDPF-treated RD cells. **D.** RD cells on the coverslips were treated with peptides (WDPF, and ΔWDPF) at the concentration of 25 μM for 2 hours, and then transfected with $2A^{pro}$ for 24 hours. The cells were fixed and stained with anti-Hsp27 (Green) and anti-hnRNPA1 (Red), followed by Alexa Fluor 488-conjugated anti-rabbit antibody and Alexa Fluor 594-conjugated anti-mouse antibody. The nuclei were stained with Hoechst (Blue). The images were captured by Nikon A1HD25 Confocal Microscope. For Hsp27, Cells with nuclear translocation were marked by white arrows. For hnRNP A1, cells with cytosol translocation were marked by white arrows. The scale bar is 20 μM. **E-F.** The JACoP-plugin of the extended ImageJ version Fiji was used to compute the M1 colocalization coefficient (the fraction of Hsp27 in Hoechst or the fraction of hnRNP A1 in Hoechst). Statistical analyses were carried out using Student’s t-test. Data are expressed as mean $\pm$SD.

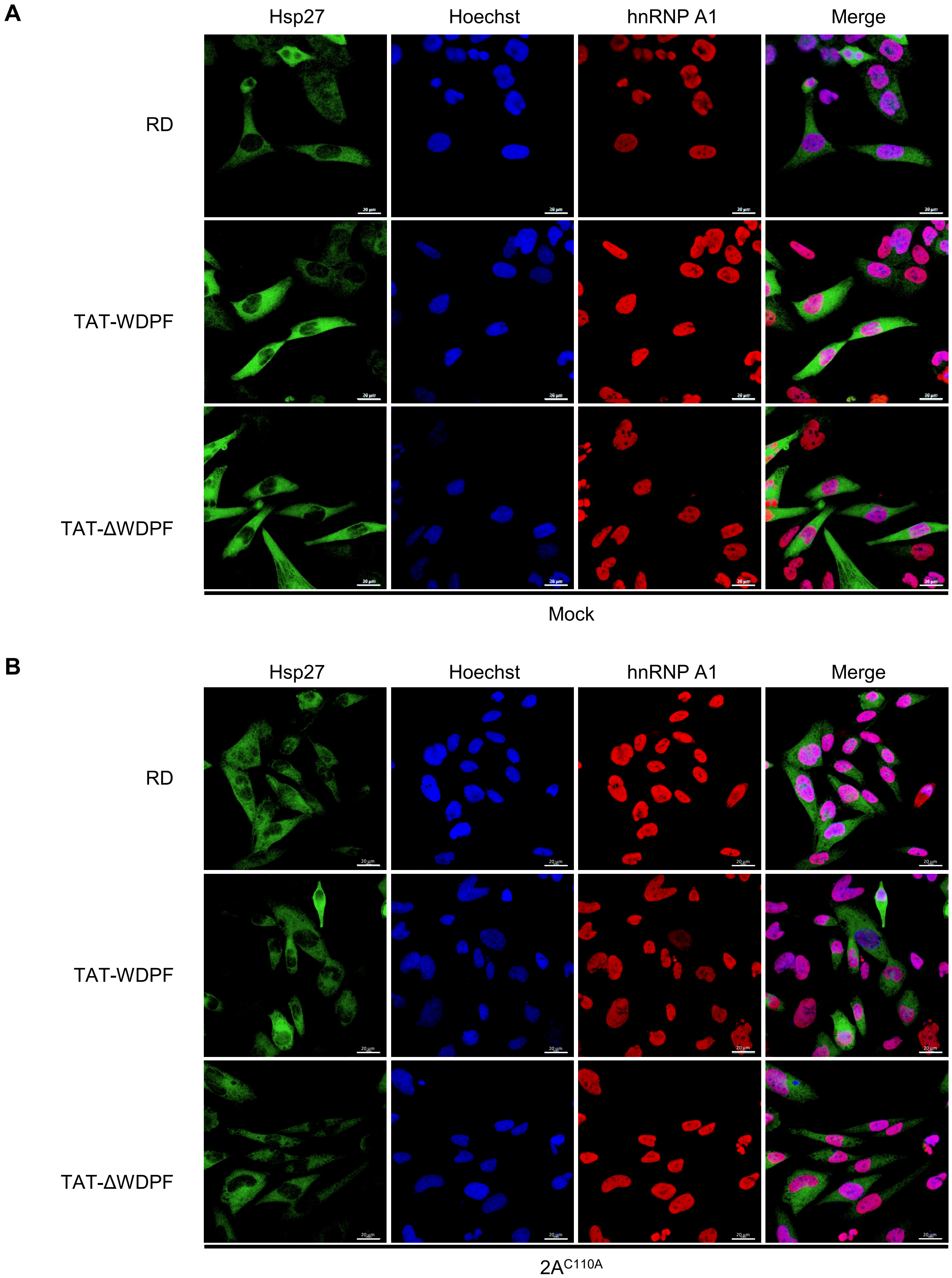

Figure S4 No effect on Hsp27/hnRNP A1 localization with or without transfection of 2A^{C110A}

A-B. RD cells on the coverslips were treated with peptides (WDPF, and ΔWDPF) at the concentration of 25 μM for 2 hours, and then transfected with vector (A) or 2A^{C110A} (B) for 24 hours. The cells were fixed and stained with anti-Hsp27 (Green) and anti-hnRNP A1 (Red), followed by Alexa Fluor 488-conjugated anti-rabbit antibody and Alexa Fluor 594-conjugated anti-mouse antibody. The nuclei were stained with Hoechst (Blue). The images were captured by Nikon A1HD25 Confocal Microscope. The scale bar is 20 μM.

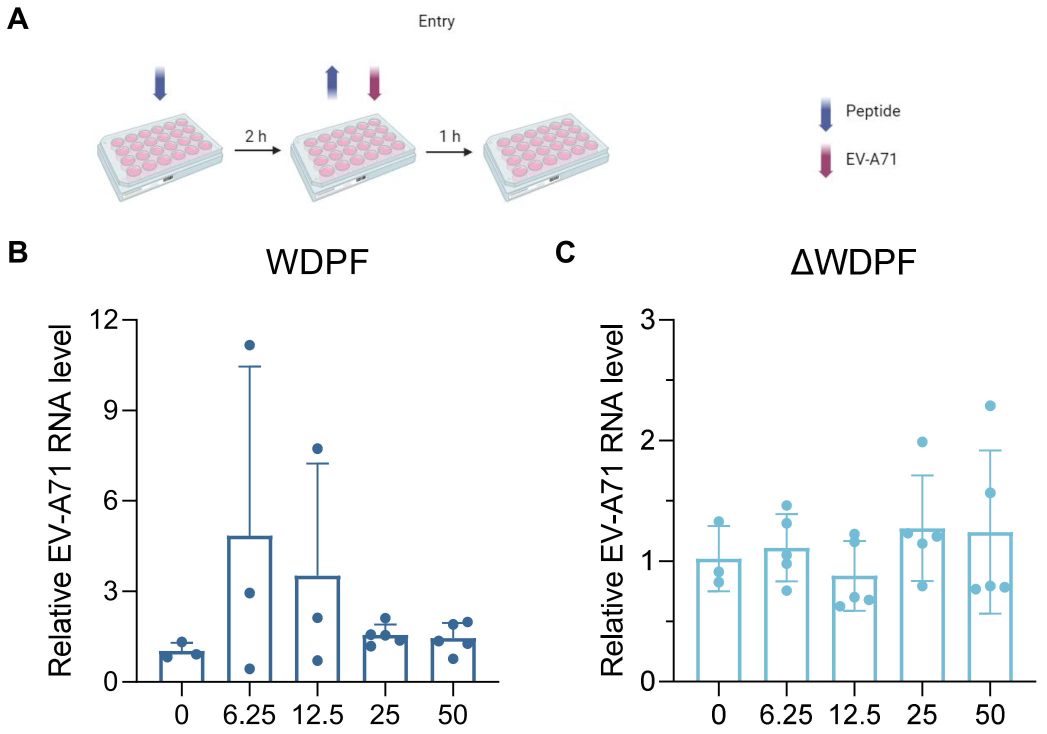

Figure S5 No effect of peptide WDPF on the entry of EV-A71

A. RD cells were pre-treated with peptides WDPF or ΔWDPF for 2 hours and infected with EV-A71 at the MOI of 1 for 1 hours. **B-C.** Intracellular viral RNA was extracted and quantified by qPCR.

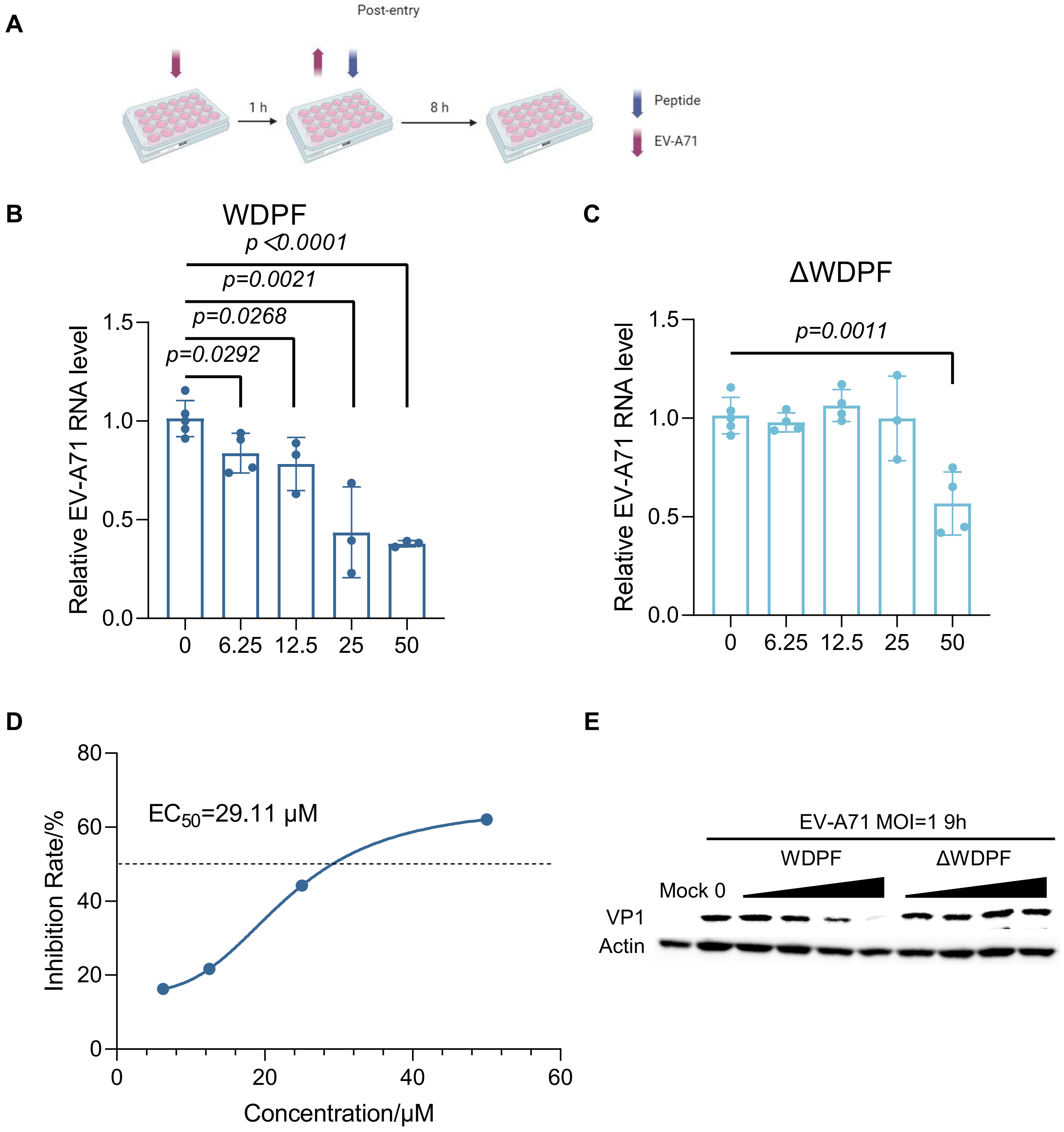

Figure S6 Inhibition of EV-A71 replication and translation at post-entry stage by peptide WDPF

A. RD cells were infected with EV-A71 at the MOI of 1 for 1 hours. Then the medium was discarded. The EV-A71-infected cells were treated with peptides WDPF or ΔWDPF for 8 hours. **B-C.** Intracellular viral RNA was extracted and quantified by qPCR. **D.** The EC_{50} of WDPF was calculated using GraphPad Prism 8.0. **E.** Cell lysates were collected for western blot assay. Statistical analyses were carried out using Student's t-test. Data are expressed as mean ±SD.

A

No TAT-WDPF (NT-WDPF)
WDPFRDWYPHSR

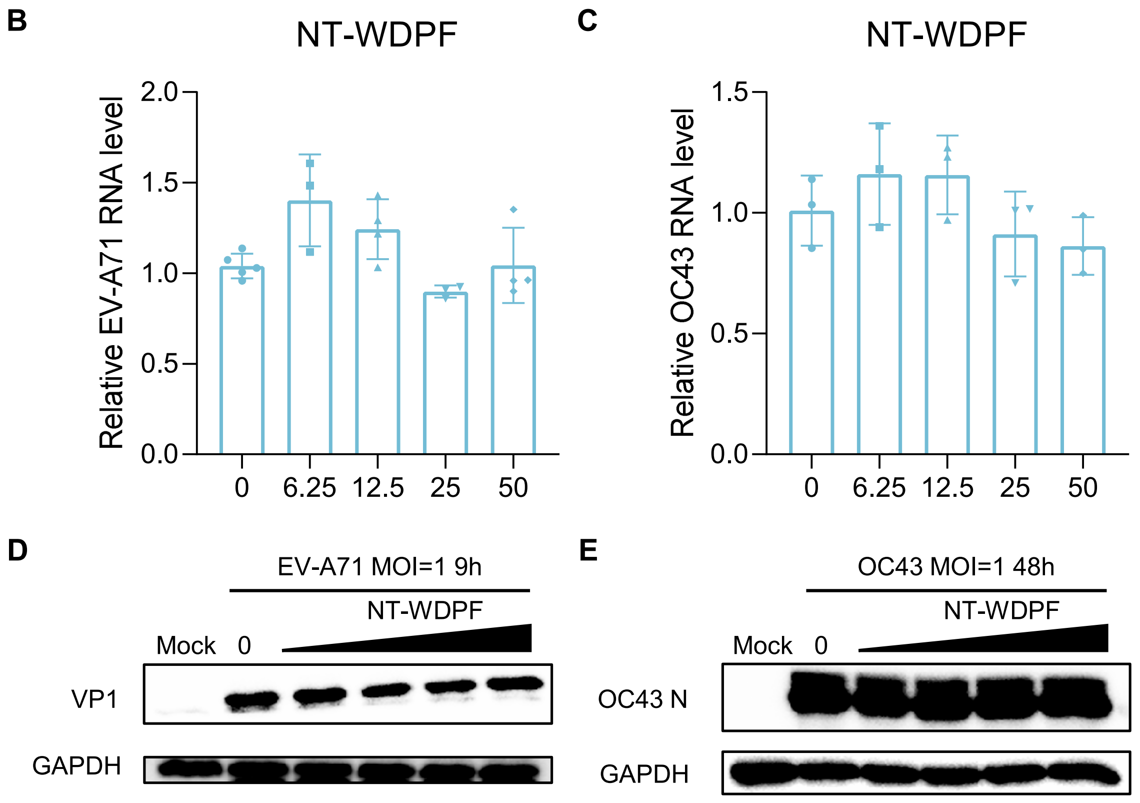

Figure S7 No effect of peptide NT-WDPF on EV-A71 and HCoV-OC43 infection

A. The amino acid sequence of peptide NT-WDPF. **B-C.** RD cells were pre-treated with peptide NT-WDPF at indicated concentrations for 2 hours and then infected with EV-A71 at the MOI of 0.1 for 24 hours or HCoV-OC43 at the MOI of 1 for 48 hours. Intracellular viral RNA was extracted and quantified by qPCR. **D-E.** Cell lysates were collected for western blot assay. The EV-A71 VP1 and HCoV-OC43 N were detected. Statistical analyses were carried out using Student's t-test. Data are expressed as mean ±SD.

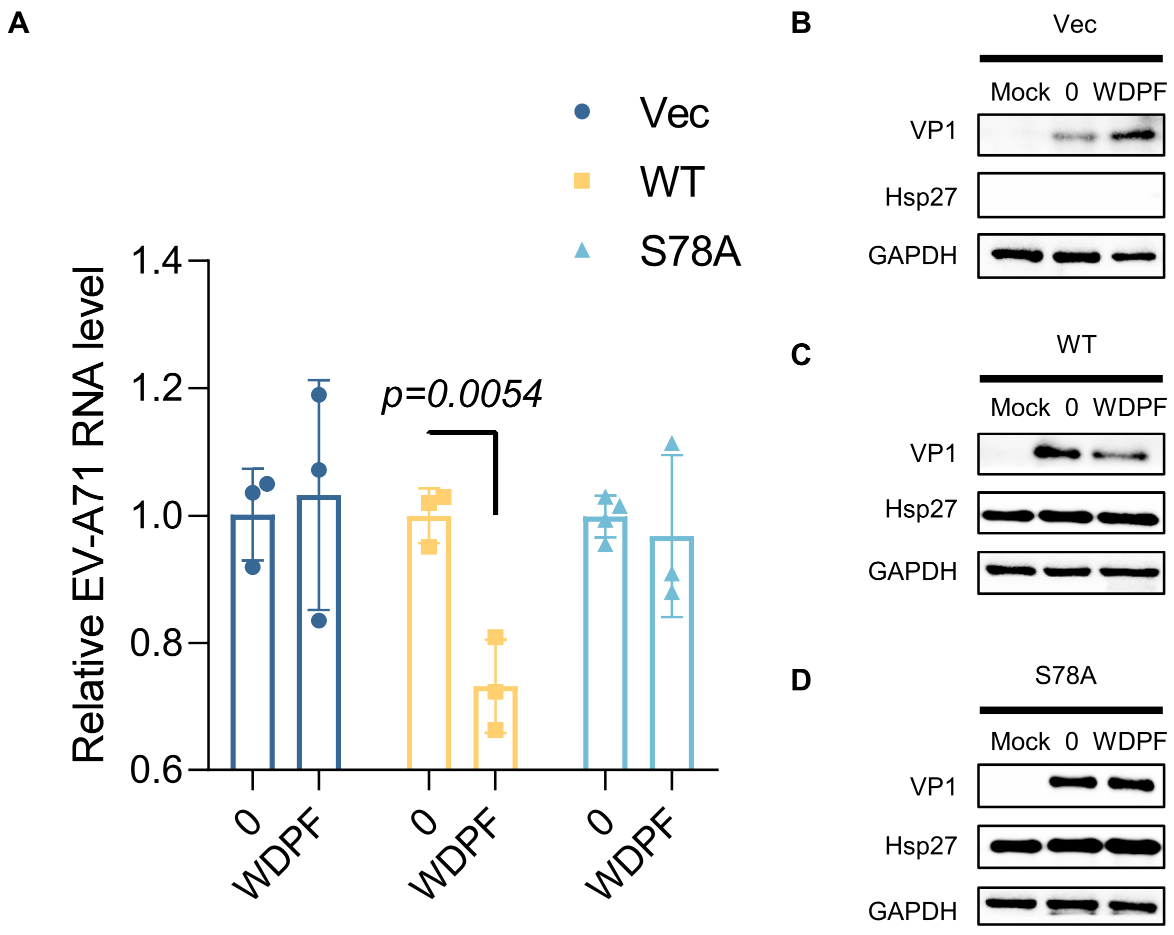

Figure S8 No effect of peptide WDPF on EV-A71-infected Hsp27-S78A-expressing cells

Hsp27 KO cells were transfected with Hsp27-WT, Hsp27-S78A, or vector plasmid (500 ng) for 24 hours. The cells were treated with peptide WDPF (50 μM) for 2 hours and then infected with EV-A71 at the MOI of 0.1 for 24 hours. **A.** Intracellular viral RNA was extracted and quantified by qPCR. **B-D.** Cell lysates were collected for western blot assay. The EV-A71 VP1 was detected. Statistical analyses were carried out using Student's t-test. Data are expressed as mean ±SD.

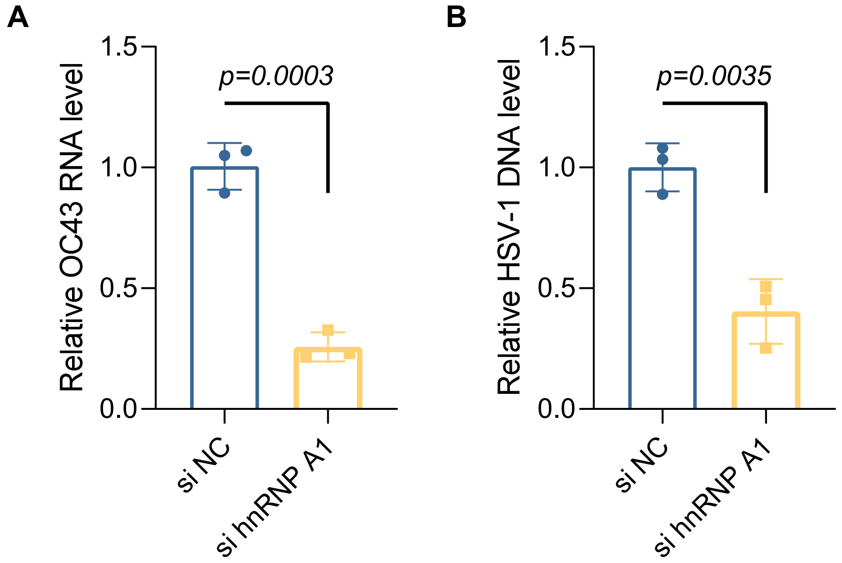

Figure S9 Inhibition of HCoV-OC43 and HSV-1 infection by knockdown of hnRNP A1

RD cells were transfected with si hnRNP A1 or si NC. **A.** The cells were then infected with HCoV-OC43 at the MOI of 1 for 48 hours. **B.** The cells were then infected with HSV-1 at the MOI of 0.1 for 24 hours. Intracellular viral RNA/DNA was extracted and quantified by qPCR. Statistical analyses were carried out using Student's t-test. Data are expressed as mean ±SD.

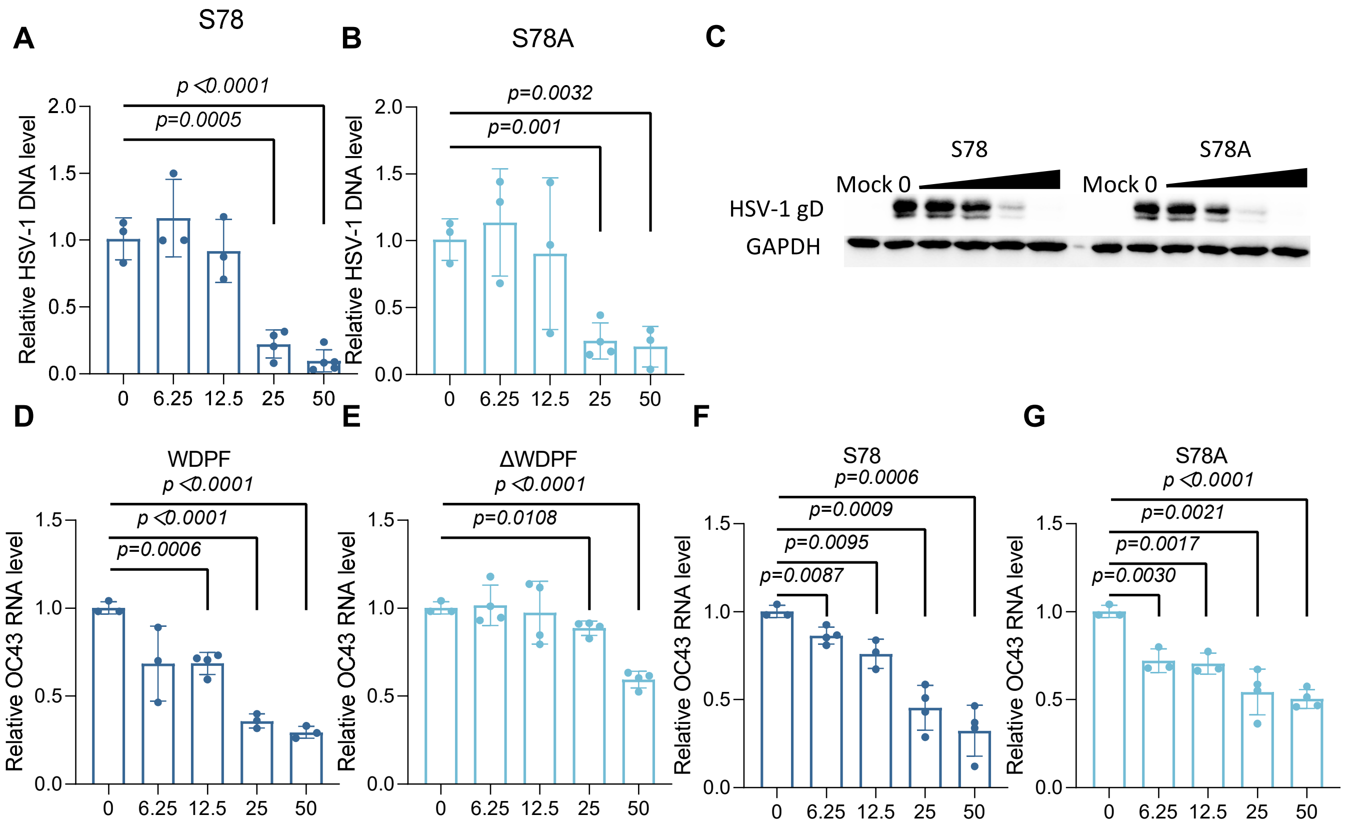

Figure S10 Inhibition of HCoV-OC43 and HSV-1 infection by peptides treatment

A-B. RD cells were treated with the indicated peptides and then infected with HSV-1 at the MOI of 0.5 for 24 hours. Intracellular viral DNA was collected and quantified by qPCR. **C.** HSV-1 gD protein were detected. **D-G.** RD cells were treated with the indicated peptides and then infected with HCoV-OC43 at the MOI of 1 for 48 hours. Intracellular viral RNA was extracted and quantified by qPCR. The EC_{50} of WDPF peptide is 20.45 μM. Statistical analyses were carried out using Student's t-test. Data are expressed as mean ±SD.

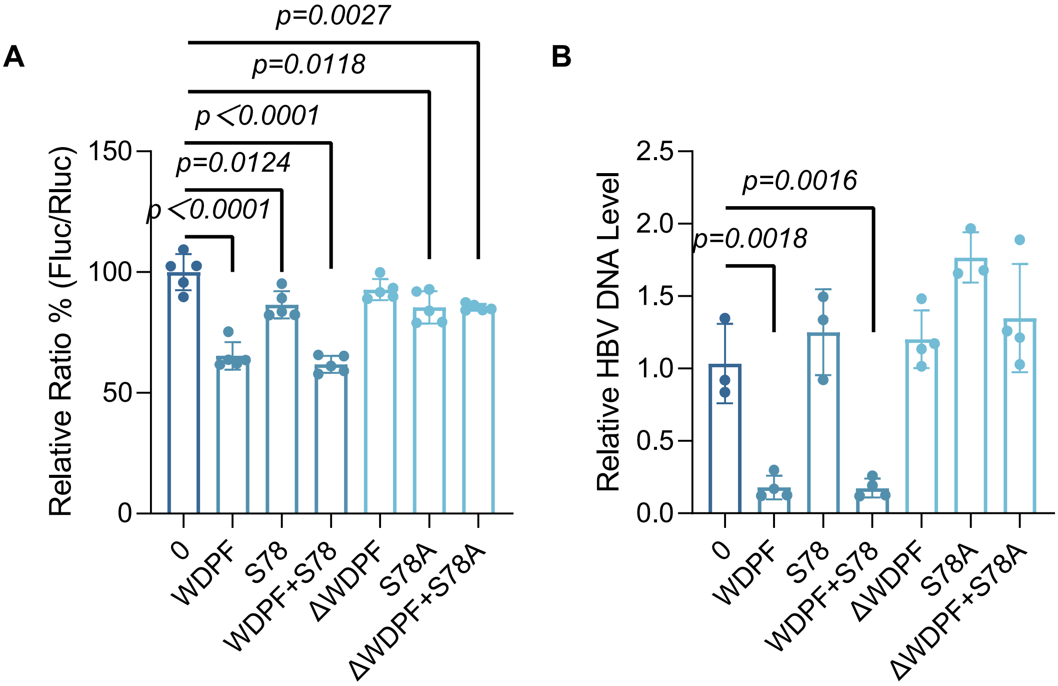

Figure S11 Inhibition of HBV infection by peptides treatment

A. 293T cells were co-transfected with Hsp27 (500 ng), the 1.3xHBV-Luciferase plasmid (200 ng) and the Renilla Luciferase Control plasmid (50 ng), and then treated with the indicated peptides (25 μM). After 24 hours, the luciferase activity was measured. **B.** 293T cells were transfected with transfected with pHBV (500 ng), and then treated with the indicated peptides (25 μM). Intracellular viral DNA was extracted and quantified by qPCR. Statistical analyses were carried out using Student's t-test. Data are expressed as mean ±SD.

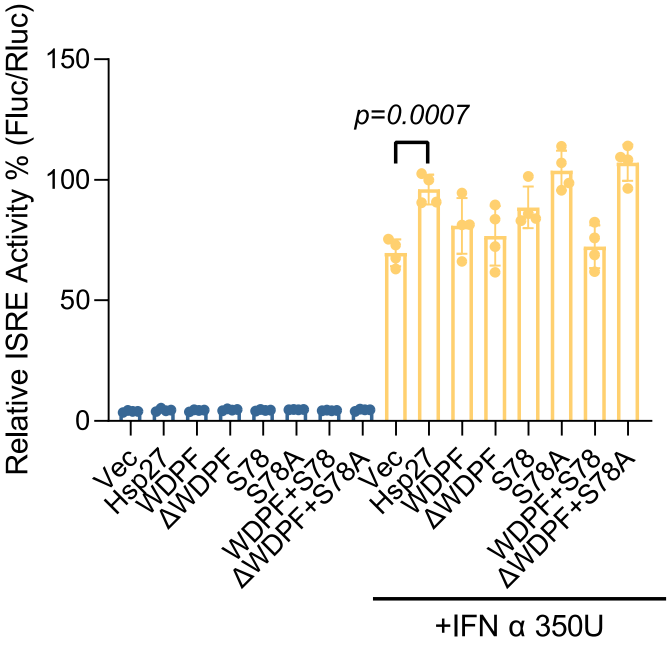

Figure S12 Peptide treatment cannot enhance IFN response

HEK 293T cells were pre-treated with peptides at the concentration of 25 μM for 2 hours and then co-transfected with vector, or Hsp27-WT plasmid (350 ng), pISRE reporter plasmid (350 ng), and pRF control reporter plasmid (50 ng). The cells were further stimulated by recombinant human IFNα (Cat#11190) for 24 h, then the luciferase activity was measured.
